# The ascorbate–glutathione cycle coming of age

**DOI:** 10.1093/jxb/erae023

**Published:** 2024-01-18

**Authors:** Christine H Foyer, Karl Kunert

**Affiliations:** School of Biosciences, College of Life and Environmental Sciences, University of Birmingham, Edgbaston B15 2TT, UK; Department of Plant and Soil Sciences, FABI, University of Pretoria, Pretoria, 2001, South Africa; Shimane University, Japan

**Keywords:** Foyer–Halliwell–Asada cycle, hydrogen peroxide, photosynthesis, redox signalling, ROS wave, superoxide, stress acclimation

## Abstract

Concepts regarding the operation of the ascorbate–glutathione cycle and the associated water/water cycle in the processing of metabolically generated hydrogen peroxide and other forms of reactive oxygen species (ROS) are well established in the literature. However, our knowledge of the functions of these cycles and their component enzymes continues to grow and evolve. Recent insights include participation in the intrinsic environmental and developmental signalling pathways that regulate plant growth, development, and defence. In addition to ROS processing, the enzymes of the two cycles not only support the functions of ascorbate and glutathione, they also have ‘moonlighting’ functions. They are subject to post-translational modifications and have an extensive interactome, particularly with other signalling proteins. In this assessment of current knowledge, we highlight the central position of the ascorbate–glutathione cycle in the network of cellular redox systems that underpin the energy-sensitive communication within the different cellular compartments and integrate plant signalling pathways.

## Teaser

The ascorbate–glutathione cycle sits at the core of plant redox regulatory networks. Ascorbate, glutathione, and reactive oxygen species not only link energy metabolism to signalling pathways but also serve a plethora of additional functions.

## Introduction

**Fig. 1.**
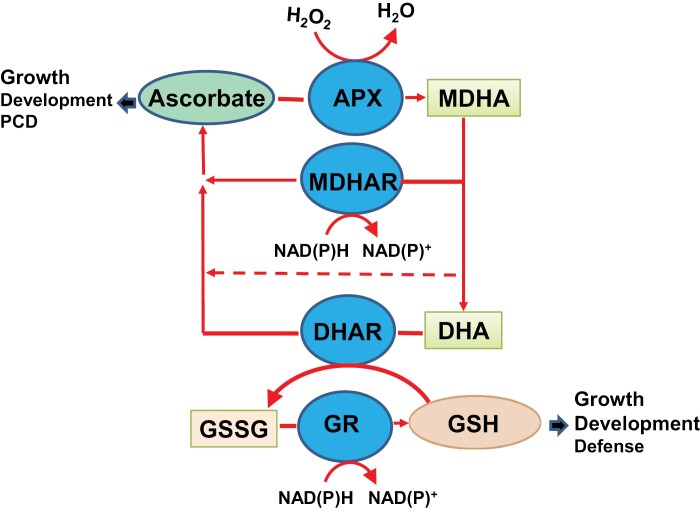
The role of the ascorbate–glutathione cycle (Asada–Halliwell–Foyer cycle) in regenerating the reduced forms of ascorbate and glutathione to maintain a wide range of biological functions. APX, ascorbate peroxidase; DHAR, dehydroascorbate reductase; GSH, reduced glutathione: GR, glutathione reductase; GSSG, glutathione disulfide; MDHA, monodehydroascorbate; MDHAR, monodehydroascorbate reductase.

It is almost 50 years since the ascorbate–glutathione cycle (sometimes called the Asada–Halliwell–Foyer cycle or Foyer–Halliwell–Asada pathway; Fig. 1) was first described in chloroplasts (Foyer and Halliwell, 1976). This was together with the proposal that the function of this cycle was to protect redox-sensitive proteins from uncontrolled oxidation by reactive oxygen species (ROS), particularly hydrogen peroxide (H_2_O_2_)_._ Thereafter, soluble ascorbate-specific peroxidases (APXs) were described for the first time (Groden and Beck, 1979; Kelly and Latzko, 1979). Intensive biochemical and molecular/genetic research efforts in the following decades demonstrated that ascorbate, glutathione, and other components of this cycle can be found in every compartment of the plant cell (Noctor and Foyer, 1998). Although the importance of antioxidants, such as ascorbate and glutathione in human diseases, had long been recognized, it was only somewhat later that the interactions between ascorbate and glutathione were considered in animal systems (Meister, 1994). Ascorbate and glutathione are the most abundant low molecular weight (LMW) antioxidants in plant cells, and their primary functions are related to interactions with ROS and other reduction/oxidation- (redox) sensitive molecules. Together with peroxiredoxins (PRXs) and thioredoxins (TRXs), the ascorbate–glutathione cycle regulates ROS accumulation in each compartment, with perhaps the exception of the apoplast/cell wall compartment (Foyer and Hanke, 2022). These PRXs reduce not only H_2_O_2_ but also alkyl hydroperoxide and peroxynitrite (Liebthal *et al.*, 2018), regulating the concentration of cellular peroxides. However, ascorbate and glutathione are multifunctional metabolites with diverse interactomes that facilitate a wide range of functions in the regulation of plant growth and development as well as defence. Moreover, each of the four component enzymes, namely APX, monodehydroascorbate reductase (MDHAR), dehydroascorbate reductase (DHAR), and glutathione reductase (GR), have subcellular isoforms that can serve different, sometimes ‘moonlighting’, functions that remain poorly characterized and understood. Hence, the action of the ascorbate–glutathione cycle extends far beyond policing ROS signals, not least because this pathway serves to maintain the reduced states of the ascorbate and glutathione pools that fulfil important but divergent roles in plant biology (Pellny *et al.*, 2009; Pasternak *et al.*, 2020; Zur *et al.*, 2021). The following discussion provides a current overview of the relevant literature, highlighting the regulation and functions of different components of the cycle, with a particular focus on signalling and regulation. We also consider possibilities for other additional functions related to the individual roles of ascorbate and glutathione.

## ROS processing and regulation of ROS signalling

Accumulating evidence suggests that ROS are essential metabolite markers, or signals of living cells (Van Breusegem *et al.*, 2018). During evolution, the management of oxygen metabolism and the associated production, accumulation, and degradation of ROS in each extracellular and intracellular compartment has become central to every aspect of biology from energy metabolism to growth, development, and defence. Superoxide and H_2_O_2_ act as either electron donors (reductants) or acceptors (oxidants). They thus engage in electron transfer (redox) processes with cellular metabolites and proteins. ROS are an integral part of the cell decision-making process in all aerobic cells, and hence overaccumulation of ROS can lead to growth arrest and cell death. However, the notion that there are ‘low’ and ‘high’ levels of ROS in plant cells that have different functions is misleading because it suggests that low ROS levels are focused on signalling while high ROS levels are involved in more negative reactions rather than signalling. In fact, all ROS molecules are potentially effective signalling molecules; no matter the level of accumulation, the capacity for signalling is limited only by the availability of interacting partners that can transfer the oxidative signal. There is little evidence that oxidative damage accumulates in plant cells to such an extent that it limits cellular functions. In many cases, ROS and oxidized lipids and proteins also function as signals that regulate gene expression to ensure appropriate acclimation or cell death responses. ROS accumulation leading to an enhanced oxidative state is a key signature of plant responses to biotic and abiotic stresses such as drought, heat, salinity, and high light (Choudhury *et al.*, 2017). Moreover, many aspects of plant development, such as the maintenance of stem cells and quiescence, and seed germination, involve an imposed ‘oxidative state’, as discussed in detail below. Each subcellular compartment in plants contains its own set of ROS-producing and ROS-scavenging pathways, but relatively little is known about how the different components in such compartmentalized systems are coordinated. Choudhury *et al.* (2017) concluded that as long as plant cells maintain high enough energy reserves to remove ROS, these essential signals are beneficial to plants during abiotic stress, enabling them to adjust their metabolism and mount a proper acclimation response. The functions of the ascorbate–glutathione cycle are powered by the pools of pyridine nucleotides, NAD(H) and NADP(H), as is ROS production by respiratory burst oxidase homologues (RBOHs) and other ROS-producing enzymes. These essential co-enzymes function as energy transducers, signalling molecules, and redox couples, the balance between the oxidized and reduced forms being important in the maintenance of cellular redox status, regulation of ion channels, and responses to environmental and metabolic challenges that determine cell fate (Noctor *et al.*, 2006).

ROS signals fulfil important roles in the regulation of numerous developmental processes from root development (Eljebbawi *et al.*, 2021; Mase and Tsukagoshi, 2021), the transition to flowering (Huang *et al.*, 2021), to leaf senescence (Zentgraf *et al.*, 2022). They contribute to the elicitation of genetic and epigenetic responses that allow acclimation and adaptation to metabolic, developmental, and environmental triggers (Ramakrishnan *et al.*, 2022). ROS and the ascorbate–glutathione cycle thereby function synchronously to regulate plant growth and development, as well as defence. For example, ROS generation is the driver and first requirement for many developmental processes, such as the cell cycle, pollen viability, microspore reprogramming towards sporophytic development, the regulation of female gametophyte patterning, and the maintenance of embryo sac polarity, as well as the prevention of self-pollonation (de Simone *et al.*, 2017; Sankaranarayanan *et al.*, 2020; Zhang *et al.*, 2021; Zur *et al.*, 2021). In such systems, the ascorbate–glutathione cycle, together with TRXs, PRXs, glutaredoxins (GRXs), and antioxidant enzymes, such as superoxide dismutase (SOD) and catalase (CAT), ensures appropriate redox-mediated regulation, so that ROS, ascorbate, and glutathione can accumulate in the required compartment-specific manner.

Cell-to-cell ROS signalling plays a pivotal role in activating local and systemic responses to environmental and developmental signals (Waszczak *et al.*, 2018; Zandalinas *et al.*, 2020, 2021; Fichman and Mittler, 2021a, b). Auto-propagating waves of ROS, calcium, and electric signals function together to generate rapid systemic cell-to-cell communication (Wang *et al.*, 2019). Succesive waves of ROS accumulation and removal are, therefore, important not only in cell-to-cell communication in plants (Zandalinas *et al.*, 2020, 2021; Fichman and Mittler, 2021a, b), but also for plant-to-plant communication (Szechyńska-Hebda *et al.*, 2022), plant–microorganism interactions (Zhou *et al.*, 2019), signalling between mammalian cells, and also in isolated animal hearts, allowing coordinated acclimation responses (Fichman *et al.*, 2023). The cell-to-cell transmission of the ROS wave can, however, be blocked by the addition of antioxidants, such as CAT or inhibitors of NADPH oxidase (also called RBOH proteins). This demonstrates that this system of cell-to-cell communication is policed by regulated production and destruction of ROS signals. The activation of RBOH proteins on the plasma membrane generates superoxide radicals in the apoplast, which are converted to H_2_O_2_, through either spontaneous dismutation or the action of SOD.

**Fig. 2.**
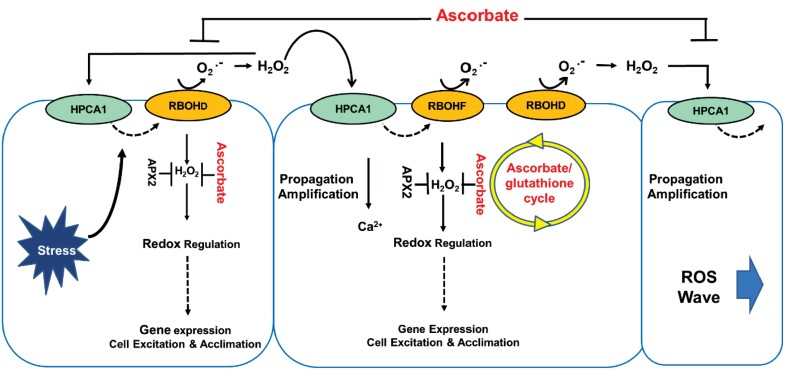
The role of antioxidants in modulating the ROS wave pathway of systemic signalling. APX2, ascorbate peroxidase2; HPCA1, HYDROGEN-PEROXIDE-INDUCED CA^2+^ INCREASES (HPCA)1; RBOHD, F, respiratory burst homologue protein D, F in *Arabidopsis thaliana*.

The process of cell-to-cell ROS signalling, which is called the ‘ROS wave’ (Fig. 2), is linked to cell-to-cell calcium and membrane potential signalling and is essential for systemic stress signalling and systemic acquired acclimation (Fichman and Mittler, 2020, 2021a, b). In this process, ROS production by the RBOHs, RBOHD and RBOHF, is triggered in the cells that are directly subjected to stress, resulting in a state of ‘activated ROS production’. The leucine-rich repeat receptor-like kinase HPCA1 (H_2_O_2_-induced Ca^2+^ increases 1) is required for coordination of ROS and calcium signals during the cell-to-cell propagation of ROS signals (Fichmann *et al.*, 2022). Once the state of activated ROS production reaches cells and tissues, other than those initiating the signal, it triggers acclimation mechanisms and enhances overall stress resilience (Fichman and Mittler, 2021a, b). Little attention has as yet been paid to how the ascorbate/gluathione cycle regulates the lifetime of ROS signals in any given cellular compartment. ROS processing and removal in activated cells is, however, an essential feature of the progression of the ROS wave.

## The ascorbate–glutathione-dependent water/water cycle and its functions

The ascorbate–glutathione cycle is comprised of metabolites (ascorbate, glutathione, and NADPH) and enzymes, which regenerate the reduced forms of ascorbate and glutathione (Fig. 1). The first step of the pathway is the reduction of H_2_O_2_ to water by the action of APX, using ascorbate acting as the electron donor. Oxidized ascorbate (monodehydroascorbate, MDA) can thereafter either spontaneously disproportionate to ascorbate and dehydroascorbate (DHA), or be reduced to ascorbate by the enzyme MDHAR, using the reducing power of NAD(P)H. In addition, the photosynthetic electron transport chain may directly reduce MDHA to ascorbate (Miyake and Asada, 1994). DHA is reduced back to ascorbate by several enzyme systems, as discussed below. However, in the classic formulation of the ascorbate–glutathione pathway (Foyer and Halliwell, 1976), DHA is reduced to ascorbate by the enzyme DHAR using reduced glutathione (GSH) as the reductant. The enzyme GR then reduces the oxidized form of glutathione, glutathione disulfide (GSSG), to GSH with NADPH as the reductant (Foyer and Halliwell, 1976).

**Fig. 3.**
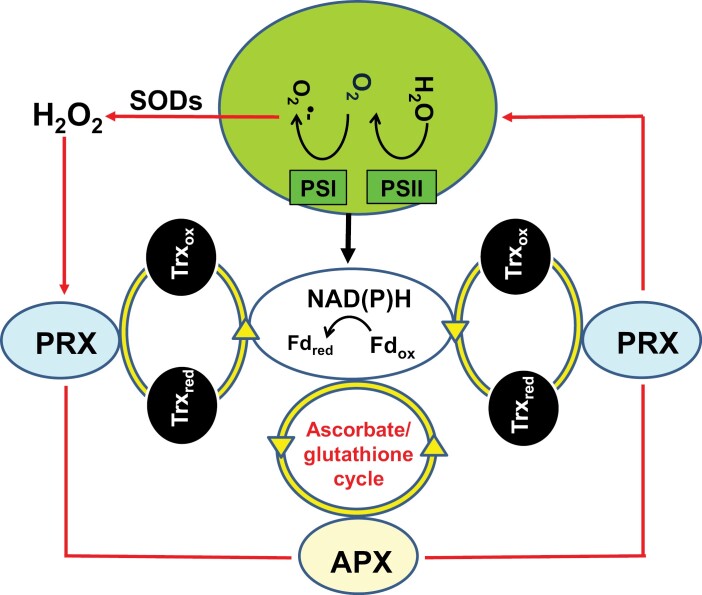
The water/water cycle allowing the dissipation of excess excitation energy and electrons providing an alternative electron sink for protection of PSII from inhibition and supporting ATP production. APX, ascorbate peroxidase; Fd_ox_, oxidized ferredoxin; Fd_red_, reduced ferredoxin; O_2_·^−^, superoxide anion radicals; PRX, 2-Cys peroxiredoxins; Trx_ox_, oxidized thioredoxin; Trx_red_, reduced thioredoxin.

The water/water cycle (WWC) is a logical extension of the activity of the ascorbate–glutathione cycle in chloroplasts (Asada, 1999), because the production and removal of H_2_O_2_ to water is coupled to the activity of the photosynthetic electron transport (PET) chain (Foyer and Hanke, 2022; Fig. 3). The water-splitting activity of PSII facilitates the transfer of elections through the PET chain to produce reduced ferredoxin and NADPH, and also produces molecular oxygen. In turn, molecular oxygen can accept electrons from many of the electron carriers in the PET chain (Foyer and Hanke, 2022), a process that is called the ‘Mehler reaction’, or ‘pseudocyclic electron flow’. The univalent reduction of oxygen by the PET chain produces superoxide (O_2_·^–^) radicals, largely at the surfaces of the thylakoid membranes. Superoxide produced on the stromal surfaces of the membranes is then rapidly converted to H_2_O_2_ by the action of thylakoid SODs. Thereafter, H_2_O_2_ is reduced to water by chloroplast APXs and the ascorbate–glutathione cycle, and also by the action of 2-Cys peroxiredoxins (PRXs). They are re-reduced either by the chloroplast TRX system (Foyer and Hanke, 2022) or by GSH and GR. Taken together, these reactions form the WWC, in which two electrons are used to produce H_2_O_2_ and two more electrons are required to metabolize H_2_O_2_ to water (Fig. 3). The WWC ultimately provides a mechanism for the dissipation of excess excitation energy and electrons, in which molecular oxygen is used as an alternative electron sink. This pathway may provide protection of PSII from photoinhibition, which still supports ATP production (Neubauer and Yamamoto, 1992). The WWC also plays a role in regulating the oxidation state of the chloroplast-targeted 2-Cys PRXs, which, together with specific atypical TRXs such as ACHT1–ACHT4 and TRXL2, are involved in the transfer of oxidative equivalents from H_2_O_2_ to target chloroplast proteins, such as those of the reductive pentose pathway (Ojeda *et al.*, 2018; Vaseghi *et al.*, 2019; Yokochi *et al.*, 2021). Similarly, the WWC plays a role in the regulation of cyclic electron flow around PSI (CEF), which serves to balance the energy budget of photosynthesis (Strand *et al.*, 2015). In this system, H_2_O_2_ functions as a signal that activates the CEF pathway, while the ascorbate–glutathione pathway serves to modulate the signal. Likewise, the primary precursor of jasmonic acid (JA), 2-oxophytodienoic acid (OPDA), interacts with 2-Cys PRX, which is suggested to act as a redox sensor through H_2_O_2_ processing and associated regulation of the TRX- and thiol-dependent regulation of enzymes of the Benson/Calvin cycle such as fructose 1,6-bisphosphatase (FBPase; Muthuramalingam *et al.*, 2013; Liebthal *et al.*, 2018). OPDA also binds to cyclophilin 20-3 (CYP20-3), which forms a complex with serine acetyltransferase 1 (SAT1). This, in turn, triggers the formation of a hetero-oligomeric cysteine synthase complex (CSC) with *O*-acetylserine(thiol)lyase B, a process that activates sulfur assimilation and the accumulation of sulfur-containing metabolites such as GSH (Watanabe *et al.*, 2021).

The 2-Cys PRXs operate through the formation of a homodimer, in which a disulfide bond connects a peroxidatic Cys (C^P^)^175^ from one monomer and is connected to the resolving Cys located on the second monomer. The oxidation of (C^P^)^175^ inhibits 2-Cys PRX activity. Reduction of the 2-Cys PRX dimers requires reductants such as GSH, TRXs, NADPH-dependent TRX reductase C (NTRC), and/or CYP20-3 (Liebthal *et al.*, 2018). GSH binding regulates the conformational state of 2-Cys PRX, favouring monomerization (Liu *et al.*, 2020). This suggests that GSH is an effective reducing agent for 2-Cys PRX that regulates the roles and functions of this redoxin in chloroplasts. Like 2-Cys PRX, other PRXs can oxidize GSH via the action of GRX (Rahantaniaina *et al.*, 2013). The active site Cys residues of Prx2 can form stable mixed disulfides with reduced GSH (Peskin *et al.*, 2016) in a glutathionylation reaction that is reversed by GRX1.

The light-dependent thiol-dependent regulation of enzymes, such as FBPase and the 46 kDa isoform of Rubisco activase, is essential for the efficient operation of photosynthesis and carbon assimilation, as well as carbon partitioning and transport. This central system of photosynthetic regulation is based on the redox modulation of Cys residues on proteins such as FBPase that have a high intrinsic reactivity. Cys residues have nucleophilic thiol side chains that are susceptible to oxidative modifications and are hence amongst the most reactive amino acids. The oxidation of Cys thiols leads to the formation of sulfenic acid (–RSOH), which can react with other reactive sulfur species to form mixed disulfides (–RSSR–) with protein thiols, *S*-glutathione adducts (–RSSG) with glutathione, or persulfides (–RSSH) with hydrogen sulfide (Willems *et al.*, 2021). The oxidation of protein Cys groups can be catalysed by enzymes, such as protein disulfide isomerases that introduce disulfide bridges during protein folding, or indirectly by thiol peroxidases via disulfide exchange reactions (Delaunay *et al.*, 2002; Veal *et al.*, 2002). These oxidation reactions are, however, reversible. The reduction reaction is catalysed by ‘redoxin’ enzymes, such as TRXs and GRXs that transfer reducing equivalents from the PET chain as well as from NAPDH, GSH, and ascorbate. Together, the ascorbate–glutathione cycle, and the redoxin systems, not only regulate but also protect protein thiols from overoxidation to sulfinic (–RSO2H) and sulfonic (–RSO3H) acids. The latter oxidation reactions are essentially irreversible and hence lead to protein inactivation. Moreover, these redox reactions form the basis for the post-translational modulation (PTM) of a wide range of proteins that regulate not only metabolism but also ROS signalling and protein–protein interactions, such as those of the chloroplast CP12-2/phosphoribulokinase (PRK)/glyceraldehyde 3-phosphate dehydrogenase (GAPB) ternary complex.

As mentioned above, the WWC is linked to OPDA signalling and GSH synthesis (Park *et al.*, 2013). Moreover, the activity of γ-glutamylcysteine synthetase (γ-ECS), which catalyses the first step of the committed GSH synthesis pathway, is regulated by oxidation, both at the level of oxidant-induced de-repression of γ-ECS translation and at the post-translational level by oxidation of enzyme thiol groups (Hicks *et al*., 2007; Noctor *et al.*, 2012). The links between the WWC, GSH, and OPDA signalling are examples of the extensive crosstalk between the redox processing systems and hormone pathways that regulate plant defence systems. Similarly, redox changes associated with the ascorbate–glutathione cycle regulate retrograde signalling from chloroplasts and mitochondria to the nucleus in order to regulate gene expression that modifies plant growth and defence responses (Mielecki *et al.*, 2020).

## Enzyme localization, properties, and functions

The enzymes of the ascorbate–glutathione cycle are localized in different intracellular compartments (Table 1). Very low levels of these enzymes have also been detected in the extracellular cell wall/apoplastic space (Vanacker *et al.*, 1998). APX1 has also been localized in the nuclei, together with SOD and CAT (Liu *et al.*, 2019; Foyer *et al.*, 2020a). Recent evidence suggests that the compartmentation of many enzymes associated with ROS processing or redox regulation is not as fixed as earlier concepts would suggest, and redox and other PTMs may facilitate re-localization of proteins to fulfil moonlighting functions (Foyer *et al.*, 2020b). The following discussion considers the enzymes of the ascorbate–glutathione cycle within this context.

**Table 1.** The subcellular localization of the enzymes of the ascorbate–glutathione cycle

Enzyme	Isoforms	Localization	Species	Reference
*APX*	APX1, APX2, (APX6) Stromal sAPX, Thylakoid tAPX APX3, (APX4)	Cytosol Chloroplast Microsomes	Arabidopsis, Sugarcane Arabidopsis Arabidopsis	Kaur *et al.* (2021) Liu *et al.* (2018) Maruta *et al.* (2010) Jardim-Messeder *et al.* (2022) Narendra *et al.* (2006)
*MDHAR*	MDHAR1, MDHAR4 MDHAR2, MDHR3 MDHAR5 MDHR6	Peroxisomes Cytosol Mitochondria Chloroplast	Arabidopsis, cotton	Lisenbee *et al.* (2005) Zhou *et al.* (2021)
*DHAR*	DHAR1 DHAR2, DHAR3	Peroxisomes? Cytosol? Cytosol Chloroplast	Arabidopsis	Terai *et al.* (2020) Rahantaniaina *et al.* (2017b)
*GR*	GR1 GR2	Cytoplasm Nucleus Peroxisomes Mitochondria Plastids	Arabidopsis	Li *et al.* (2022) Delorme-Hinoux *et al.* (2016) Amr *et al.* (2010) Marty *et al.* (2019)

The APXs are haem-containing enzymes that belong to class I of the peroxidase–catalase superfamily (Lazarotto *et al.*, 2021). APXs are encoded by small gene families, with different isoforms targeted to the cytosol, plastids, mitochondria, and peroxisomes (Lazarotto *et al.*, 2021). Some APX forms are associated with membranes, such as the plasmalemma, the peroxisomal membranes, and the thylakoid membranes, often together with MDHARs, while other APX forms are in the soluble phase. The APX forms differ in substrate affinities, dimer formation, and the presence of transmembrane domains. Moreover, the cytosolic APX of *Oncidium* orchid (*Og*cytAPX1) uses GSH as a substrate as well as ascorbate, but with different active sites (Chin *et al.*, 2019). The Pro63, Asp75, and Tyr97 residues are required for GSH oxidation by *Og*cytAPX1, whereas the corresponding site in *At*APX1 is composed of Asp63, His75, and His97, and has no GSH binding activity. In addition to *Og*cytAPX1, the recombinant cytosolic APX forms from maize, rice, and soybean also possess GSH oxidation activity (Chin *et al.*, 2019). Such interactions, like those linking GSH to the reduction of 2-Cys PRX, demonstrate that there are multiple additional levels of complexity to the ascorbate–glutathione cycle.

Some APX forms, such as Arabidopsis *At*APX1 (Kaur *et al.*, 2021) and the rice *Os*APX2 (Hong *et al.*, 2018), have chaperone functions. However, only the high molecular weight (HMW) complexes of *At*APX1 and *Os*APX2 display chaperone activity, whereas the LMW forms exhibit predominantly PRX activity (Hong *et al.*, 2018). These APX forms undergo structural and functional transitions between HMW and LMW forms. In addition, certain APX isoforms are highly sensitive to oxidative inactivation (Shikanai *et al.*, 1998). Hence, 2-Cys PRXs and other PRXs are required to ensure H_2_O_2_ processing in organelles, such as chloroplasts that produce large amounts of this oxidant.

Like other enzymes of the ascorbate–glutathione cycle, APXs are also subjected to PTMs. For example, the peroxidase activity of APX1 is regulated by *S*-nitrosation, tyrosine nitration, and *S*-sulfhydration either negatively or positively, depending on the plant species (Begara-Morales *et al.*, 2016; Aroca *et al.*, 2018). The Arabidopsis APX1 protein has five Cys residues, of which two (Cys32 and Cys49) are *S*-nitrosated (Yang *et al.*, 2015). The Cys32 residue is also the target for *S*-sulfhydration (Aroca *et al.*, 2015), which could regulate the binding affinity of APX1 for ascorbate, resulting in increased PRX activity. Tyrosine nitration has been also suggested to inhibit APX1 activity in pea and tobacco (Clark *et al.*, 2000; Begara-Morales *et al.*, 2013). Moreover, protein phosphorylation catalysed by the calcium-dependent protein kinase, CPK 28, activates APX2 activity through phosphorylation at Thr59 and Thr164 (Hu *et al.*, 2021). In contrast, a wheat kinase, called start 1.1, translocates to chloroplasts where it binds and phosphorylates tAPX, decreasing its activity and ability to remove H_2_O_2_ (Gou *et al.*, 2015). Crotonylation of protein Lys residues is an important PTM that has been recently shown to regulate many plant processes (Contreras-de la Rosa *et al.*, 2022). For example, crotonylation of Lys136 in the chrysanthemum APX increases enzyme activity to increase protection against low-temperature stress (Lin *et al.*, 2021).

Nitric oxide (NO) is an important regulator of ROS accumulation in plants through the regulated enhancement of the activities of ROS-scavenging enzymes, such as APX, CAT, and SOD, for example during stress responses (Beligni *et al.*, 2002; Xue *et al.*, 2007; Keyster *et al.*, 2011; Begara-Morales *et al.*, 2014). In the presence of molecular oxygen, NO undergoes an *S*-nitrosation reaction with GSH, forming GSNO, which leads to PTMs and nitration of proteins, such as APX (Correa-Aragunde *et al.*, 2015). While NO inhibits the activity of the cytosolic APX in tobacco Bright Yellow-2 suspension cells through *S*-nitrosation (de Pinto *et al.*, 2013), *S*-nitrosation positively regulates the activity of the Arabidopsis cytosolic APX1, upon exposure to stress (Yang *et al.*, 2015), and contributes to the suppression of cell death responses (Lin *et al.*, 2011). NO also regulates H_2_O_2_ levels and hence the shelf life and nutritional quality of pepper fruits through modulation of the different APX isozymes (González-Gordo *et al.*, 2022). NO reacts with O_2_^−^ to produce peroxynitrite (ONOO^−^), a molecule that can nitrate lipids, nucleic acids, aromatic rings, and the tyrosine residues in proteins leading to tyrosine nitration. This selective PTM can regulate enzyme activity, as well as preventing or promoting tyrosine phosphorylation.

MDHARs are typical FAD monomeric enzymes that catalyse redox reactions using FADH as substrate to reduce MDHA to ascorbate (Zhou *et al.*, 2021). MDHAR activity is crucial for enhancing the efficiency of the APX reaction in cellular compartments where the activities of these enzymes are coupled. MDHARs have been divided into three classes: class I, chloroplastic/mitochondrial enzymes; class II, peroxisomal membrane-attached enzymes; and class III, cytosolic/peroxisomal enzymes (Tanaka *et al.*, 2021). All plants have class II and III enzymes, which are the peroxisomal membrane-attached and cytosolic/peroxisomal isoforms, while some plants lack class I chloroplastic/mitochondrial enzymes. The chloroplast MDHAR forms are activated by TRXs. For example, the plastidial MDHAR form is activated by TRXy2, and the activity of a recombinant plastid Arabidopsis MDHAR isoform (MDHAR6) increases in the presence of reduced TRXy, and not other plastidial TRXs (Vanacker *et al.*, 2018).

In addition to MDHA, MDHARs can also use organic radicals as substrates (Hossain *et al.*, 1984). MDHARs recycle the oxidation products of other powerful antioxidants, such as phenolic compounds: ferulic acid, quercetin, chlorogenic acid, and coniferyl alcohol (Sakihama *et al.*, 2000). MDHAR6 reacts, for example, with the explosive 2,4,6-trinitrotoluene (TNT), generating superoxide (Johnston *et al.*, 2015). Plasma membrane electron transport from ascorbate to MDHA has also been proposed, in a process that involves a high-potential plant plasma membrane cytochrome *b* (Horemans *et al.*, 1994). Moreover, NO scavenging by barley haemoglobin is facilitated by the MDHAR-mediated ascorbate reduction of methaemoglobin (Igamberdiev *et al.*, 2006).

The overexpression of *MDHAR* genes has consistently been shown to increase ascorbate accumulation and increase plant stress tolerance (Eltayeb *et al.*, 2007; Kavitha *et al.*, 2010; Li *et al.*, 2010; Yin *et al.*, 2010; Eltelib *et al.*, 2012; Yeh *et al.*, 2019). In contrast, mutants lacking MDHAR do not always show changes in ascorbate accumulation. For example, the peroxisomal membrane-associated ascorbate-dependent electron transfer system involves APX as well as MDHAR. While the Arabidopsis peroxisomal membrane APX isoform (APX3) is dispensable for growth and development (Narendra *et al.*, 2006), the seedling-lethal *sugar-dependent2* mutant is deficient in the peroxisomal membrane MDHAR isoform (MDHAR4). MDHAR4 mutants also have lower ascorbate to DHA ratios, but have similar total ascorbate levels to the wild type (Eastmond, 2007). Taken together, these findings suggest that other system enzymes, in addition to MDHAR, may not be a rate-limiting step in ascorbate recycling.

The DHAR enzymes belong to the glutathione *S*-transferase (GST) superfamily and have a characteristic two-domain architecture, comprised of a mixed α/β N-terminal domain containing the glutaredoxin motif (CXX[C/S]) and an all-helical C-terminal domain (Littler *et al.*, 2010). The active site comprises a glutathione-binding G-site and a hydrophobic substrate-binding H-site. The reaction probably proceeds via a ‘ping–pong’ mechanism, where DHA binds to the free reduced form of DHAR followed by binding of GSH (Ding *et al.*, 2020).

The requirement and functions of the DHARs in ascorbate regeneration have, however, long been a matter of debate (Morell *et al.*, 1997, 1998; Foyer and Mullineaux, 1998). Genetic studies using DHAR overexpression, knockdown, and/or knockout lines supported the physiological importance of DHARs in ascorbate recycling (Chen *et al.*, 2003; Chen and Gallie, 2004, 2006, 2008; Gallie, 2013; Noshi *et al.*, 2016, 2017). For example, the multivitamin white corn variety with high DHAR activity has a 6-fold higher kernel ascorbate level than controls (Naqvi *et al.*, 2009). Moreover, DHAR gene expression is also associated with enhanced abiotic stress tolerance (Broad *et al.*, 2020). Loss‐of‐function mutations in the Arabidopsis cytosol-targeted DHAR2 form alone led to lower ascorbate/DHA ratios but did not affect total ascorbate accumulation (Yoshida *et al.*, 2006). Nevertheless, the physiological role of DHARs remains uncertain, largely because the Arabidopsis triple-knockout (*dhar1 dhar2 dhar3*) mutants that lack all three DHARs have negligible DHAR activity and display similar levels of ascorbate to the wild-type controls, with ascorbate/DHA ratios as well as plant growth and development similar to the wild type (Rahantaniaina *et al.*, 2017a, b). In addition, the absence of DHAR activity had no impact on the ascorbate profiles of the catalase-deficient mutant (*cat2*) that maintains a highly oxidized glutathione pool. DHAR activity was also required for the GGSG accumulation and cell death phenotypes that are observed in the *cat2* mutants under stress conditions (Rahantaniaina *et al.*, 2017a). Moreover, DHAR activity was required to maintain ascorbate recycling capacity under high light conditions in the *phytoalexin*-*deficient 2*-*1* (*pad2*-*1*) mutants that have low glutathione accumulation (Terai *et al.*, 2020). Hence, multiple systems including MDHAR, DHAR, glutathione, and ferredoxin contribute to the generation of reduced ascorbate. For example, the CPYC-type GRXs exhibit DHAR activity (Sha *et al.*, 1997; Rouhier *et al.*, 2003). Other as yet uncharacterized proteins may also have DHAR activity (Morell *et al.*, 1997). Nevertheless, current evidence suggests that GSH is required for ascorbate regeneration under high light conditions (Terai *et al.*, 2020). DHAR activity also maintained the ascorbate pool in mutants that have low ascorbate accumulation (Terai *et al.*, 2020), and other recycling systems contribute to ascorbate recycling when ascorbate levels are high.

GRs are responsible for maintaining the cellular glutathione pools in the reduced state. As such, these flavoprotein oxidoreductases are crucial regulators of plant development and the responses to environmental stress (Foyer *et al.*, 1995; Noctor *et al.*, 1998). GSH is a central signalling molecule in plants that functions together with the GRX and TRX systems to regulate numerous phytohormone-associated pathways (Rai *et al.*, 2023). It also serves as a cofactor for various enzymes, such as GRXs and GSTs, which play crucial roles in cell detoxification pathways. A recent study also proposed that GSH, together with neodiosmin, is a signature metabolite for pattern-triggered immunity and effector-triggered immunity involving surface-localized pattern recognition receptors and intracellular nucleotide-binding leucine-rich repeat receptors (Lu *et al.*, 2023).

Higher plant GRs are encoded by two genes: *GR1* and *GR2*. While *GR1* encodes a cytosolic, or peroxisomal, form of the enzyme, *GR2*, which contains a long N-terminal sequence, encodes a mitochondrial and chloroplastic form. The chloroplast form represents ~80% of the total GR activity. GR has also two Cys residues that form a redox-active disulfide bridge at the active site. Glutathione disufide binds to the active site to form a disulfide bond separately with a Cys residue and a His residue at the active site allowing reduction to GSH (Kataya and Reumann, 2010; Yousuf *et al.*, 2012; Couto *et al.*, 2016). Overexpression of the chloroplast form of GR significantly increases the GSH content of plants and increases tolerance to a range of abiotic stresses (Foyer *et al.*, 1995; Li *et al.*, 2005; Gill *et al.*, 2013). The chloroplast-localized GR2 also fulfils essential roles in root apical meristem maintenance (Yu *et al.*, 2013).

GRXs are thioltransferases that serve a number of important roles in plants (Meyer *et al.*, 2008, 2012, 2021). These small (12 kDa) redox enzymes catalyse not only the reduction of disulfides, but also the reduction of mixed disulfides, in a process called deglutathionylation. Hence, they act as oxidoreductases that control glutathionylation/deglutathionylation reactions. GRX functions depend on two distinct interaction sites for efficient redox catalysis (Begas *et al.*, 2017). The first site interacts with the GSH moiety of glutathionylated disulfide substrates. The second site activates GSH as the reducing agent (Begas *et al.*, 2017). There are five GRX subgroups that are classified according to their active site sequences, of which groups III and IV are specific to vascular plants. In *Arabidopsis thaliana*, group I proteins that have C[P/G/S]Y[C/S] in the catalytic site are localized in the cytosol and plastids and are encoded by six genes. The group I GRXs undertake oxidoreductase functions and are found in most organisms. The four group II (monothiol GRXs or CGFS GRXs) GRXs in *A. thaliana* are localized in the cytosol, plastid, nucleus, and mitochondria. The third type of GRXs have a Cys-Cys-X-Cys or Cys-Cys-X-Ser sequence at the active site and are specific to higher plants. They are also called ROXY GRXs (Zaffagnini *et al.*, 2008; Ströher and Millar, 2012). There are 21 members of group III in *A. thaliana* that are localized in the cytoplasm and nucleus. Group IV proteins have a GRX domain followed by four CxxC repeats at the C-terminus (Navrot *et al.*, 2006). Group V (CPF[C/S]) has six members that are found in the cytosol, mitochondria, and chloroplast.

Class II GRXs act as iron–sulfur (Fe–S) cluster bridging proteins. They function as maturation factors for the production of Fe–S proteins (Rey *et al.*, 2019). As such, they participate in iron homeostasis and the maturation of Fe–S protein [2Fe–2S] clusters with interacting proteins. For example, the GRX [2Fe–2S] clusters form complexes with BOLA proteins, in which the [2Fe–2S] cluster is ligated using the GRX conserved Cys, a Cys from GSH, and His or Cys residues on the BOLA protein. The function of the plastid GRXs as Fe–S cluster bridging proteins links them to the thylakoid membrane functions and chlorophyll metabolism. Like PRXs and TRXs, the GRX proteins may serve functions in organelle to nucleus retrograde signalling pathways (Sevilla *et al.*, 2023).

## Moonlighting functions

Many of the proteins involved in the ascorbate–glutathione cycle reside in different intracellular compartments where they can have ‘moonlighting’ as well as enzymatic functions. APXs have a broad substrate specificity and possess chaperone activity, hence participating in various biological processes (Li, 2023). Of the eight *AtAPX* genes in *A. thaliana*, three encode cytosolic (cytAPXs: *At*APX1, 2, and 6) proteins, three microsomal/peroxisomal (perAPXs: *At*APX3, 4, and 5) proteins, and two chloroplastic (chlAPXs: soluble stromal *At*sAPX and thylakoid membrane-bound *At*tAPX) protein isoforms (Panchuk *et al.*, 2005; Granlund *et al.*, 2009). Like the rice *Os*APX2 protein (Hong *et al.*, 2018), *At*APX1 has chaperone functions (Kaur *et al.*, 2021). The LMW forms of *At*APX1 and *Os*APX2 exhibit peroxidase activity, but the HMW complexes also display chaperone activity.

The *At*APX4 and *At*APX6 (APX-L and APX-R) proteins lack essential catalytic residues, ASC-binding sites, and haem-binding sites (Granlund *et al.*, 2009). These proteins, which are generally encoded by a single gene, have been reclassified as two novel families of class I peroxidases (Lazzarotto *et al.*, 2015). The chloroplast-targeted *At*APX6 protein is also found in the cytosol and functions as a haem peroxidase that does not use ascorbate as a substrate to reduce H_2_O_2_ (Lazzarotto *et al.*, 2021). APXs can also oxidize non-physiological aromatic substrates *in vitro*, such as *p*-cresol, *o*-dianisidine, and guaiacol, at rates comparable with ascorbate (Raven, 2003). For example, the soluble cytosolic coumarate 3-hydroxylase (C3H) enzymes of *A. thaliana* and *Brachypodium distachyon* can oxidize both ascorbate and 4-coumarate at comparable rates (Barros *et al.*, 2019).

No moonlighting functions have as yet been reported for MDHAR proteins, which can reduce a wide range of substrates in addition to DHA. However, the class II enzymes attach to the peroxisomal membrane and have essential roles in plant development (Eastmond, 2007). The *A. thaliana At*MDAR4 protein, which binds to the peroxisomal membrane, protects the SDP1 triacylglycerol lipase from oxidation, but the mechanistic reasons for this phenotype are unknown. The *sdp2* mutants that lack the class II *At*MDHAR4 enzyme have a seedling-lethal phenotype in the absence of exogenous sugar treatment. The siliques of *mdar1-2*^*(+/−)*^*mdar4-5*^*(−/−)*^ double mutants have both normal and empty seeds, whereas those of the wild type and single mutants have only normal seeds, suggesting that the double knockout of both isoforms causes embryonic lethality (Tanaka *et al.*, 2021).

Plant DHARs are dimorphic proteins that exist in soluble enzymatic and membrane-integrated forms. They share a structural glutathione *S*-transferase (GST) fold with human chloride intracellular channels (HsCLICs). HsCLICs are dimorphic proteins that exist in soluble enzymatic and membrane-integrated ion channel forms. *At*DHAR1 is able to generate inward conductance in transfected mammalian cell membranes (Das *et al.*, 2016) and the *Pennisetum glaucum* (*Pg*)DHAR is dimorphic and has been localized in the plasma membrane (Das *et al.*, 2023). Thus, DHAR can function as an oxidative stress-regulated ion channel (Das *et al.*, 2023).

## Support for ascorbate functions in plants

Ascorbate is a multifunctional metabolite (Table 2) that regulates plant growth and development (Foyer *et al.*, 2020b). It is a major non-enzymatic antioxidant and ubiquitous ROS scavenger that is better (>100× faster) than GSH at scavenging superoxide and singlet oxygen. As such, it interacts with various redox regulatory signalling networks and plays a key role in redox signal transduction, particularly in relation to abiotic stress tolerance. For example, ascorbate was found to have a specific and light-dependent effect on the expression of the gene encoding the chloroplast 2-Cys peroxiredoxin-A2, an effect that could not be substituted by GSH (Shaikhali and Baier, 2016). The concentration of ascorbate in *A. thaliana* cells is the lowest in the vacuoles (2.3 mM), with higher levels in the mitochondria (10.4 mM), chloroplasts (10.8 mM), and nuclei (16.3 mM). The highest ascorbate concentrations were found in the cytosol (21.7 mM) and peroxisomes (22.8 mM) (Zechmann, 2011; Zechmann *et al.*, 2011). In comparison, the concentrations of ascorbate (0.002 mM) and DHA (0.36 mM) in the apoplast are relatively low (Booker *et al.*, 2012).

**Table 2.** The functions of ascorbate in plants

Function	Target	Reference
ROS processing	Removal of superoxide and hydrogen peroxide (e.g. produced in photosynthesis; Arabidopsis)	Awad *et al.* (2015); Karpinska *et al.* (2017); Foyer *et al.* (2020)
Antioxidant regeneration	α-Tocopherol reduction	Munne-Bosch (2005)
Electron donor/acceptor	(PSI/PSII) (e.g. barley, Arabidopsis)	Mano *et al.* (2004); Tóth *et al.* (2009)
Enzyme cofactor	Peroxidase substrate (e.g. ascorbate peroxidase; poplar) De-epoxidation (violaxanthin de-epoxidase; rice) Hydroxylation (Fe- and 2-oxoglutarate-dependent dioxygenases; ethylene, GA, ABA) (e.g. tomato, rice, Arabidopsis) Flavonoid biosynthesis (Arabidopsis)	Miyake and Asada (1994); Mehlhorn *et al.* (1996) Müller-Moulé *et al.* (2002); Saga *et al.* (2010) Wei *et al.* (2021); Smirnoff (2018); Bulley *et al.* (2021); Ye and Zhang (2012) Page *et al.* (2012)
*Enzyme inhibitor*	Chloroplast antioxidant enzyme (Arabidopsis)	Horling *et al.* (2003)
*Flower development*	Anther/pollen development (e.g. orchid, Arabidopsis)	Deslous *et al.* (2021)

Ascorbic acid can be as efficient as SOD in catalysing the removal of superoxide radical (Som *et al.*, 1983). The rate constant for the reaction between ascorbic acid and superoxide (at pH 7.4) using the xanthine–xanthine oxidase system was estimated to be 5.4 × 10^6^ M^–1^ s^–1^ (Som *et al.*, 1983). However, Gray and Carmichael (1992) reported that the rate constant for bovine erythrocyte Cu,Zn-SOD was *k*SOD=6.4 × 10^9^ M^–1^ s^–1^ which is 1000 times higher. Nevertheless, the lifetime of superoxide as a signalling molecule can be considered to depend on the presence of SODs and ascorbate, which essentially police this molecule. Superoxide accumulation in plant stem cells such as those found in the shoot apical meristem (SAM) and the root apical meristem (RAM) is important in defining the identity of undifferentiated meristematic cells (Tsukagoshi *et al.*, 2010; Zeng *et al.*, 2017). Like SODs, ascorbate is largely absent from the quiescent centre of the RAM. The addition of ascorbic acid stimulates not only the activity of the quiescent centre cells but also cell proliferation in the entire root meristem (Liso *et al.*, 1998).

Ascorbate may also play a key role in policing organelle to nucleus communication and signalling pathways. For example, mutations in proteins such as the rice chloroplast-localized pseudouridine synthase (OSPUS 1-1) lead to the production of albino seedlings under low temperatures because of aberrant chloroplast ribosome biogenesis (Wang *et al.*, 2022). Overexpression of mitochondrial MnSOD also rescues the phenotype, as does the suppressor protein of *ospus 1-1*, which encodes a mitochondrial pentapeptide repeat (PPR) protein. Such findings suggest that there is coordinated superoxide signalling between the mitochondria and chloroplasts that regulates plastid development. The chloroplast ascorbate–glutathione system, particularly the chloroplast APXs, has been found to regulate signalling related to stress experiences, such as low temperature stress, over time without the requirement of establishing cold acclimation (van Buer *et al.*, 2016). Moreover, cold priming was found to modify chloroplast to nucleus signalling by thylakoid APX-mediated suppression of CEF mediated by the thylakoid NADH dehydrogenase complex (Seiml-Buchinger *et al.*, 2022).

Dry seeds are devoid of reduced ascorbate and APX activity. They contain only DHA, suggesting that the ascorbate–glutathione cycle does not function in dry seeds. Clearly the reduced ascorbate content of plant organs has to be maintained within certain thresholds, according to tissue requirements. Attempts to enhance ascorbate levels must therefore be approached with caution because artificially high ascorbate levels as a consequence of removing feedback controls were shown to impair reproductive development (Deslous *et al.*, 2021).

Ascorbate is also an essential enzyme cofactor that participates in the regulation of photosynthesis and metabolism. It is a specific cofactor for a large family of enzymes known as the Fe- and 2-oxoglutarate-dependent dioxygenases that catalyse the addition of a hydroxyl group to various substrates (Wei *et al.*, 2021). Ascorbate is required for the maintenance of activity of Fe(II)/2-oxoglutarate-dependent dioxygenases via reduction of Fe(III). As such, ascorbate is involved in the synthesis of phytohormones and secondary metabolites. For example, ascorbate is required for opening the ring structure of 1-aminocyclopropane-1-carboxylic acid (ACC) by supplying the electron to the active site of ACC oxidase, which catalyses the last step of ethylene biosynthesis (Smirnoff, 2018). Ascorbate has also been implicated in auxin catabolism and the synthesis of abscisic acid and gibberellins through its functions with different dioxygenases.

Ascorbate is the natural substrate for many types of plant peroxidases (Mehlhorn *et al.*, 1996). In this way, ascorbate influences the accumulation of a wide range of phenolic compounds, particularly in the cell wall/apoplastic compartment of plant cells. Ascorbate regulates the expression of genes involved in flavonol and anthocyanin precursor synthesis (Page *et al.*, 2012) such as PHENYLALANINE AMMONIA-LYASE1 (PAL1), 4-COUMARATE:COENZYME A LIGASE3, CHALCONE SYNTHASE (CHS), as well as the MYB transcription factor PAP1 and an ELONGATED HYPOCOTYL5 (HY5) homologue HYH (Munné-Bosch *et al.*, 2013). The low levels of leaf ascorbate in ascorbate-deficient mutants (*vtc2-1* and *vtc2-4*) causes, however, a significant decrease in leaf anthocyanin contents (Plumb *et al.*, 2018).

Leaf ascorbate accumulation is modulated by the amount and quality of light. Leaf ascorbate accumulation is lowest at night and highest at the end of the day. Similarly, increases in the light red/far red ratios (a ‘shade’ phenotype) resulted in much lower leaf ascorbate and GSH contents than high red/far red ratios (Bartoli *et al.*, 2009; Foyer *et al.*, 2020b). Blue light has been shown to activate the expression of the gene encoding GDP-l-galactose phosphorylase (GGP), which is the main controlling step of the l-galactose pathway of ascorbate synthesis (Bournonville *et al.*, 2023). This protein resides in the cytoplasm and the nucleus, where it interacts with the PAS/LOV photoreceptor protein (PLP) to mediate light-dependent control of ascorbate synthesis. PLP is a non-competitive inhibitor of GGP that is inactivated upon exposure to blue light (Bournonville *et al.*, 2023). Light increases APX, MDHAR, and GR activities. Light-dependent regulation of APX and MDHAR activities of these enzymes occurs via PTMs as well as at the level of gene expression (Gulyás *et al.*, 2023).

The Arabidopsis *vtc2/vtc5* double mutants, which are unable to synthesize ascorbate, are not viable (Dowdle *et al*., 2007). Mutants that have a low ascorbate content have significant reprogramming of gene expression, including genes involved in hormone synthesis and signalling, as well as photosynthesis and defence (Kiddle *et al.*, 2003; Pastori *et al.*, 2003). These changes are accompanied by increases in the levels of salicylic acid (SA), pathogenesis-related proteins, and camalexin that demonstrate the activation of the ROS signalling branch of plant innate immunity (Pavet *et al.*, 2005; Mukherjee *et al.*, 2010). In this way, ascorbate can exert a key role in plant immunity, as well as defence responses to abiotic environmental stresses (Pastori *et al.*, 2003; Pavet *et al.*, 2005; Venkatesh and Park, 2014; Akram *et al.*, 2017) including salt stress (Shalata *et al.*, 2001). Ascorbate accumulation is also important in the regulation of plant defences against biotrophic pathogens that rely on SA signalling such as *Pseudomonas syringae* and *Peronospora parasitica* (Pavet *et al.*, 2005; Mukherjee *et al.*, 2010) as well as phloem-feeding insects (Kerchev *et al.*, 2013). In contrast, ascorbate deficiency enhances susceptibility to the necrotrophic pathogen *Alternaria brassicicola*, in which defence is mediated by jasmonic acid and ethylene signalling (Botanga *et al.*, 2012). The application of exogenous ascorbate also acts as an inducer of disease resistance in plant interactions with different types of pathogens including viruses (Fujiwara *et al.*, 2013). The mechanisms involved in such strategies are complex, because reduced ascorbate is highly susceptible to oxidation in aqueous solution and, moreover, it is likely to be oxidized by the ascorbate oxidase activities in the apoplast/cell wall compartments of the plant cell before it enters the cytoplasm. The role of ascorbate in programmed cell death (PCD) is related to its role in the control of the activation of the ROS signalling branch of innate immune responses (Pavet *et al.*, 2005; Mukherjee *et al.*, 2010) Localized PCD, similar to that occurring during hypersensitive responses to plant pathogens, is observed in the leaves of ascorbate-deficient mutants (Pavet *et al.*, 2005). Increased ascorbate synthesis, resulting from supplying l-galactono-1,4-lactone, delays PCD during kernel maturation in durum wheat, with a consequent postponement of dehydration and improvement in kernel filling (Paradiso *et al.*, 2012).

Ascorbate may also influence plant epigenetic processes (Ramakrishnan *et al.*, 2022; Seiml-Buchinger *et al.*, 2022). Ascorbate is a cofactor for the ten−eleven translocation (TET1−TET3) family of proteins in mammalian cells, which are responsible for the removal of cytosine methylation in DNA (Zhithovich, 2020). Ascorbate drives the active removal of this transcription-repressive mark by enhancing the activities of TET enzymes. The TET enzymes are Fe(II)-dependent dioxygenases that catalyse a series of consecutive oxidations of 5-methylcytosine. No TET-like enzymes have as yet been identified in plants, although 5-methylcytosine oxidation products, particularly 5-hydroxymethylcytosine (5hmC), have been found in plants (Mahmood and Dunwell, 2019). However, superoxide may influence the activities of proteins that contain the [Fe–S] clusters that mediate the regulation of DNA demethylation in a manner that is regulated by ascorbate.

Ascorbate fulfils a number of important roles in the regulation of photosynthesis, particularly in the acclimation of plants to high light (Müller-Moulé *et al.*, 2014; Karpinska *et al.*, 2017). In addition to its participation in the WWC, ascorbate is also required for the regeneration of lipid-soluble antioxidants, particularly tocopherols and tocotrienols (vitamin E), which protect the polyunsaturated fatty acids in the thylakoid membranes from oxidation to chromanoxyl radicals by singlet oxygen. These radicals are converted back to vitamin E by the reducing power of ascorbate, or by reaction with carotenoids. Ascorbate is also required for the conversion of violaxanthin to zeaxanthin in the light-dependent xanthophyll cycle, which is a key component of the thermal energy dissipation mechanisms measured by the non-photoenergy quenching component of Chl *a* fluorescence (Müller-Moulé *et al.*, 2002). Knockout mutants of the chloroplast envelope ascorbate transporter *At*PHT4;4 are compromised in thermal energy dissipation (Miyaji *et al.*, 2015). Moreover, ascorbate is a potent specific inhibitor of the expression of 2-Cys PRX A and other chloroplast antioxidant enzymes (Horling *et al.*, 2003; Baier *et al.*, 2004). This influences chloroplast to nucleus signalling pathways via the redox-sensitive transcription factor Rap2.4a (Shaikhali *et al.*, 2008). Conversely, the expression of chloroplast APX and MDHAR is induced in lines defective in 2-Cys PRXs (Baier *et al.*, 2000). Ascorbate is finally also able to donate, as well as accept, electrons from the PET chain, acting as an alternative electron donor for PSII (Mano *et al.*, 2004; Tóth *et al.*, 2009).

While ascorbate has been largely discounted as a significant factor in NO metabolism (Wang and Hargrove, 2013), the ascorbate-mediated regulation of flowering in plants, such as in *Oncidium*, acts through the NO-mediated flowering-repression pathway (Kumar *et al.*, 2016). Arabidopsis low ascorbate mutants have long been known to show early flowering (Barth *et al.*, 2006), a trait that is linked to the altered expression of genes, such as flowering locus T (*FT*) and CONSTANS (*CO*) that regulate flowering (Kotchoni *et al.*, 2009). Similar effects on flowering have been reported for plants with altered APX or ascorbate oxidase (AO) activities (Pnueli *et al.*, 2003; Pignocchi *et al.*, 2006). Moreover, the exogenous application of ascorbate or its precursor l-galactono-1,4-lactone delays flowering (Shen *et al.*, 2009).

## Support for glutathione functions

Reduced glutathione (γ-glutamyl-cysteinyl-glycine: GSH) is one of the most abundant LMW non-protein thiols in plants. GSH reacts with superoxide and H_2_O_2_, but this reaction is relatively slow compared with ascorbate (Winterbourn, 2016). Nevertheless, GSH is an essential metabolite with a wide range of important functions in plant biology (Noctor *et al.*, 2012; Hasanuzzaman *et al.*, 2017; Aslam *et al.*, 2021; Dorion *et al.*, 2021; Dumanović *et al.*, 2021). The glutathione redox couple (GSH/GSSG) functions together with other redox-active couples, such as NADPH/NADP^+^ and TRX-SH/TRX-SS, to maintain cellular redox homeostasis and propagate redox signals (Foyer and Noctor, 2011; Considine and Foyer, 2021; Le Gal *et al.*, 2021).

GR activity ensures that plant cells maintain very high GSH:GSSG ratios. Decreases in GSH:GSSG ratios stimulate the reversible formation of mixed disulfides between protein sulfhydryl groups and GSSG (i.e. *S*-glutathionylation), as well as GSH synthesis. *S*-Glutathionylation of proteins results in structural and functional modifications in redox-sensitive enzymes, that can, for example, regulate PET and plant immune responses (Grek *et al.*, 2013). The 2-Cys PRX proteins are glutathionylated by GSSG, a process that favours dimerization and inactivates their molecular chaperone activities (Park *et al.*, 2011). OPDA signalling also modulates GSH-dependent protein glutathionylation in a manner that regulates PET efficiency, as well as defence gene expression.

GPXs are, therefore, considered as part of the glutathione/ascorbate cycle. Plant GPX protein sequences have high sequence similarities to mammalian phospholipid hydroperoxide GPX4 (Faltin *et al.*, 2010), containing three conserved non-selenium Cys residues at the active sites. However, they catalyse the reduction of H_2_O_2_ using TRX and GRX as the electron donor rather than GSH. They are, therefore, more correctly called thiol peroxidases than GPXs (Bela *et al.*, 2015). The plant GPX protein family consists of multiple isoenzymes located in different subcellular compartments that have distinct expression patterns with respect to tissues and developmental stages (Gao *et al.*, 2014). These enzymes play an important role in protection against environmental stress (Zhang *et al.*, 2019). For example, transgenic plants overexpressing GPX genes have better stress tolerance (Diao *et al.*, 2014; Zhang *et al.*, 2019). Some GSTs also have GPX activity. These enzymes can detoxify lipid hydroperoxides and thus participate in antioxidative defence (Dixon *et al.*, 2005; Ding *et al.*, 2020). Plant GSTs are finally mostly cytosolic enzymes, and they can represent up to 2% of soluble proteins (Pascal and Scalla, 1999).

As discussed above, GRXs play important but non-overlapping roles in iron trafficking and the biogenesis of iron-containing cofactors (Berndt *et al.*, 2021). For example, GRX17 is required for the maturation of cytosolic and nuclear Fe–S proteins, with both foldase and a redox-dependent holdase functions in cluster biogenesis that are important for stress tolerance (Martins *et al.*, 2020). GRXs participate in the regulation of plant growth and development, as well responses to environmental triggers. For example, the class III GRXS3/4/5/8 proteins function downstream of cytokinins in Arabidopsis to negatively regulate primary root growth in response to nitrate (Patterson *et al.*, 2016). These GRXs mediate cytokinin-dependent responses, acting downstream of type-B response regulators that mediate the transcriptional responses to cytokinin to inhibit root growth in response to high nitrogen^.^ (Patterson *et al.*, 2016). In particular, AtGRXS8 represses the transcriptional and developmental responses of the primary root to nitrate, by interfering with the activity of the TGA1 and TGA4 transcription factors (Ehrary *et al.*, 2020).

GSH interacts with NO, forming *S*-nitrosoglutathione, which can sequester iron in LMW compounds named mono- and dinitrosyl iron complexes. GSNO functions as a mobile reservoir of NO, which is regulated in cells by the activity of GSNO reductases that modulate NO levels in plant cells (Sakamoto *et al.*, 2002; Corpas *et al*, 2013). Protein *S*-nitrosation is reversed by TRXs and *S*-nitrosoglutathione reductases (glutathione-dependent formaldehyde dehydrogenases). GSH works together with TRXs in a range of other processes, such as the redox control of PCD. A thiol-redox switch mechanism involving TRX and GSH mediates the propagation of apoptosis signals and acts as a redox checkpoint in mammalian cells (Benhar, 2020) In this system, the nitration of various proteins controls PCD in a manner that is reversed by TRX and GSH (Benhar, 2020).

## Conclusions and perspectives

While a major function of the ascorbate–glutathione cycle is the policing of H_2_O_2_ signalling in the different subcellular compartments and also the intensity of the cell-to-cell ROS signalling wave, it also maintains the essential and multifaceted functions of ascorbate and GSH in plants. For example, ascorbate and GSH support the activities of different enzyme systems that fulfil important functions in plant growth and development. Moreover, ascorbate functions as a much more efficient superoxide scavenger than GSH and hences polices superoxide-dependent activities and signalling. The diverse functions of ascorbate and glutathione in plant biology require that the enzymes of the ascorbate–glutathione cycle do not always operate in synchrony. Clearly, the reduction of MDHA and DHA does not always require GSH, particularly in compartments in which the reduction of these metabolites by other systems is rapid, such as occurs in the vicinity of the PET chain. Similarly, the transport systems for the reduced and oxidized forms of ascorbate and glutathione facilitate the exchange of these metabolites between different compartments in a manner that remains poorly characterized. Thus, a number of factors including competing reactions, and the regulation of metabolite synthesis, degradation, and compartmentation determine whether GSH turnover is coupled to ascorbate turnover. The factors that integrate the pathways of ascorbate and GSH synthesis, recycling, and degradation remain poorly understood, although the compartmentation of these different processes is likely to be an important control point. While our understanding of the regulation of the enzymes of the pathway has greatly increased, some aspects such as the moonlighting functions remain to be fully elucidated.

While the functions of the ascorbate–glutathione cycle are well characterized in some organelles such as the chloroplasts and peroxisomes, virtually nothing is known about the roles of ascorbate and glutathione in the nucleus. Accumulating evidence suggests that superoxide and H_2_O_2_ are produced in the nucleus, where they fulfil important regulatory functions (Diaz-Vivancos *et al*., 2015; [Bibr CIT0045][Bibr CIT0075]). For example, superoxide accumulation is required to maintain shoot meristem cells and the undifferentiated meristematic cells in the root (Zeng *et al.*, 2017; Zhao *et al.*, 2023, Preprint). Little is known about how the levels of superoxide are regulated to maintain cell fate within the stem cell niche, but modulation of SOD and the ascorbate–glutathione cycle are important in the control of this system. In particular, the roles of superoxide and SOD in plant nuclei are poorly documented. In breast cancer cells, acetylation converts SOD2 from a mitochondrial antioxidant to a nuclear histone demethylase to promote cell stemness and promotes cancer cell evolution (Coelho *et al.*, 2022). In this situation, FeSOD functions as a H3 histone demethylase that requires H_2_O_2_ as a substrate (Coelho *et al.*, 2022). The nuclei of plant cells are rapidly oxidized in response to stresses, such as high temperatures (Babbar *et al.*, 2021).

The metabolites and proteins that contribute to ROS production in the nucleus remain to be identified. However, the direct impacts of stress-induced oxidation of nuclei have significant implications for current concepts of redox sensing and regulation, as well as associated signal transduction pathways (Sevilla *et al.*, 2023). The control of nuclear thiol–disulfide redox states by nucleoredoxins and TRX1 remains, however, largely uncharacterized (Kneeshaw *et al.*, 2017). Similarly, how the nuclear glutathione and ascorbate pools influence the regulation of cell cycle proteins is also still not clear (Diaz-Vivancos *et al.*, 2015; de Simone *et al.*, 2017).

In conclusion, the ascorbate–glutathione cycle sits at the heart of redox biology and interacts on multiple levels with the wider network of oxidants, ROS processing proteins, and antioxidants that regulate every aspect of plant biology. There is now a huge literature on the ascorbate–glutathione cycle, including new and important findings that add context and complexity to cycle functions. The wider significance of the ascorbate–glutathione cycle is only now becoming apparent, as new signalling mechanisms, systems, and pathways are identified.

## Data Availability

This manuscript does not contain original data

## References

[CIT0001] Akram NA , Shafiq F, Ashraf M. 2017. Ascorbic acid—a potential oxidant scavenger and its role in plant development and abiotic stress tolerance. Frontiers in Plant Science 8, 613. 28491070 10.3389/fpls.2017.00613 PMC5405147

[CIT0002] Amr R , Kataya A, Reumann S. 2010. Arabidopsis glutathione reductase 1 is dually targeted to peroxisomes and the cytosol. Plant Signaling & Behavior 5, 171–175. 20038819 10.4161/psb.5.2.10527 PMC2884127

[CIT0003] Aroca A , Gotor C, Romero LC. 2018. Hydrogen sulfide signaling in plants: emerging roles of protein persulfidation. Frontiers in Plant Science 9, 1369. 30283480 10.3389/fpls.2018.01369 PMC6157319

[CIT0004] Aroca A , Serna A, Gotor C, Romero LC. 2015. S-sulfhydration: a cysteine posttranslational modification in plant systems. Plant Physiology 168, 334–342. 25810097 10.1104/pp.15.00009 PMC4424021

[CIT0005] Asada K. 1999. The water–water cycle in chloroplasts: scavenging of active oxygens and dissipation of excess photons. Annual Review of Plant Physiology and Plant Molecular Biology 50, 601–639. 10.1146/annurev.arplant.50.1.601 15012221

[CIT0006] Aslam S , Gul N, Mir MA, Asgher M, Al-Sulami N, Abulfaraj AA, Qari S. 2021. Role of jasmonates, calcium, and glutathione in plants to combat abiotic stresses through precise signaling cascade. Frontiers in Plant Science 12, 668029. 34367199 10.3389/fpls.2021.668029 PMC8340019

[CIT0007] Awad J , Stotz HU, Fekete A, Krischke M, Engert C, Havaux M, Berger S, Mueller MJ. 2015. 2-Cysteine peroxiredoxins and thylakoid ascorbate peroxidase create a water–water cycle that is essential to protect the photosynthetic apparatus under high light stress conditions. Plant Physiology 167, 1592–1603. 25667319 10.1104/pp.114.255356 PMC4378167

[CIT0008] Babbar R , Karpinska B, Grover A, Foyer CH. 2021. Heat-induced oxidation of the nuclei and cytosol. Frontiers in Plant Science 11, 617779. 33510759 10.3389/fpls.2020.617779 PMC7835529

[CIT0009] Baier M , Noctor G, Foyer CH, Dietz KJ. 2000. Antisense suppression of 2-cysteine peroxiredoxin in Arabidopsis specifically enhances the activities and expression of enzymes associated with ascorbate metabolism but not glutathione metabolism. Plant Physiology 124, 823–832. 11027730 10.1104/pp.124.2.823 PMC59186

[CIT0010] Baier M , Ströher E, Dietz KJ. 2004. The acceptor availability at photosystem I and ABA control nuclear expression of 2-Cys peroxiredoxin-A in *Arabidopsis thaliana*. Plant and Cell Physiology 45, 997–1006. 15356325 10.1093/pcp/pch114

[CIT0011] Barros J , Escamilla-Trevino L, Song LH, et al. 2019. 4-Coumarate 3-hydroxylase in the lignin biosynthesis pathway is a cytosolic ascorbate peroxidase. Nature Communications 10, 1994. 10.1038/s41467-019-10082-7 PMC6491607 31040279

[CIT0012] Barth C , De Tullio M, Conklin PL. 2006. The role of ascorbic acid in the control of flowering time and the onset of senescence. Journal of Experimental Botany 57, 1657–1665. 16698812 10.1093/jxb/erj198

[CIT0013] Bartoli CG , Tambussi EA, Fanello D, Foyer CH. 2009. Control of ascorbic acid synthesis and accumulation and glutathione by the incident light red/far red ratio in *Phaseolus vulgaris* leaves. FEBS Letters 583, 118–122. 19059408 10.1016/j.febslet.2008.11.034

[CIT0014] Begara-Morales JC , Chaki M, Sanchez-Calvo B, Mata-Perez C, Leterrier M, Palma JM, Barroso JB, Corpas FJ. 2013. Protein tyrosine nitration in pea roots during development and senescence. Journal of Experimental Botany 64, 1121–1134. 23362300 10.1093/jxb/ert006 PMC3580824

[CIT0015] Begara-Morales JC , Sánchez-Calvo B, Chaki M, Valderrama R, Mata-Pérez C, López-Jaramillo J, Padilla MN, Carreras A, Corpas FJ, Barroso JB. 2014. Dual regulation of cytosolic ascorbate peroxidase (APX) by tyrosine nitration and S-nitrosylation. Journal of Experimental Botany 65, 527–538. 24288182 10.1093/jxb/ert396 PMC3904709

[CIT0016] Begara-Morales JC , Sánchez-Calvo B, Chaki M, Valderrama R, Mata-Pérez C, Padilla MN, Corpas FJ, Barroso JB. 2016. Antioxidant systems are regulated by nitric oxide-mediated post-translational modifications (NO-PTMs). Frontiers in Plant Science 7, 152. 26909095 10.3389/fpls.2016.00152 PMC4754464

[CIT0017] Begas P , Liedgens L, Moseler A, Meyer AJ, Deponte M. 2017. Glutaredoxin catalysis requires two distinct glutathione interaction sites. Nature Communications 8, 14835. 10.1038/ncomms14835 PMC5382279 28374771

[CIT0018] Bela K , Horvath E, Galle A, Szabados L, Tari I, Csiszar J. 2015. Plant glutathione peroxidases: emerging role of the antioxidant enzymes in plant development and stress responses. Journal of Plant Physiology 176, 192–201. 25638402 10.1016/j.jplph.2014.12.014

[CIT0019] Beligni MV , Fath A, Bethke PC, Lamattina L, Jones RL. 2002. Nitric oxide acts as an antioxidant and delays programmed cell death in barley aleurone layers. Plant Physiology 129, 1642–1650. 12177477 10.1104/pp.002337 PMC166752

[CIT0020] Benhar M. 2020. Oxidants, antioxidants and thiol redox switches in the control of regulated cell death pathways. Antioxidants 9, 309. 32290499 10.3390/antiox9040309 PMC7222211

[CIT0021] Berndt C , Christ L, Rouhier N, Mühlenhoff U. 2021. Glutaredoxins with iron–sulphur clusters in eukaryotes—structure, function and impact on disease. Biochimica et Biophysica Acta 1862, 148317. 32980338 10.1016/j.bbabio.2020.148317

[CIT0022] Booker FL , Burkey KO, Jones AM. 2012. Re-evaluating the role of ascorbic acid and phenolic glycosides in ozone scavenging in the leaf apoplast of *Arabidopsis thaliana* L. Plant, Cell & Environment 35, 1456–1466. 10.1111/j.1365-3040.2012.02502.x PMC4864724 22380512

[CIT0023] Botanga CJ , Bethke G, Chen Z, Gallie DR, Fiehn O, Glazebrook J. 2012. Metabolite profiling of *Arabidopsis* inoculated with *Alternaria brassicicola* reveals that ascorbate reduces disease severity. Molecular Plant-Microbe Interactions 25, 1628–1638. 23134520 10.1094/MPMI-07-12-0179-R

[CIT0024] Bournonville C , Mori K, Deslous P, et al. 2023. Blue light promotes ascorbate synthesis by deactivating the PAS/LOV photoreceptor that inhibits GDP-l-galactose phosphorylase. The Plant Cell 35, 2615–2634. 37052931 10.1093/plcell/koad108 PMC10291033

[CIT0025] Broad RC , Bonneau JP, Hellens RP, Johnson AAT. 2020. Manipulation of ascorbate biosynthetic, recycling, and regulatory pathways for improved abiotic stress tolerance in plants. International Journal of Molecular Sciences 21, 1790. 32150968 10.3390/ijms21051790 PMC7084844

[CIT0026] Bulley SM , Cooney JM, Laing W. 2021. Elevating ascorbate in Arabidopsis stimulates the production of abscisic acid, phaseic acid, and to a lesser extent auxin (IAA) and jasmonates, resulting in increased expression of DHAR1 and multiple transcription factors associated with abiotic stress tolerance. International Journal of Molecular Sciences 22, 6743. 34201662 10.3390/ijms22136743 PMC8269344

[CIT0027] Chen Z , Gallie DR. 2004. The ascorbic acid redox state controls guard cell signaling and stomatal movement. The Plant Cell 16, 1143–1162. 15084716 10.1105/tpc.021584 PMC423206

[CIT0028] Chen Z , Gallie DR. 2006. Dehydroascorbate reductase affects leaf growth, development, and function. Plant Physiology 142, 775–787. 16891549 10.1104/pp.106.085506 PMC1586046

[CIT0029] Chen Z , Gallie DR. 2008. Dehydroascorbate reductase affects non-photochemical quenching and photosynthetic performance. Journal of Biological Chemistry 283, 21347–21361. 18539599 10.1074/jbc.M802601200

[CIT0030] Chen Z , Young TE, Ling J, Chang SC, Gallie DR. 2003. Increasing vitamin C content of plants through enhanced ascorbate recycling. Proceedings of the National Academy of Sciences, USA 100, 3525–3530. 10.1073/pnas.0635176100 PMC152326 12624189

[CIT0031] Chin DC , Senthil Kumar R, Suen CS, Chien CY, Hwang MJ, Hsu CH, Xuhan X, Lai ZX, Yeh KW. 2019. Plant cytosolic ascorbate peroxidase with dual catalytic activity modulates abiotic stress tolerances. iScience 16, 31–49. 31146130 10.1016/j.isci.2019.05.014 PMC6542772

[CIT0032] Choudhury FK , Rivero RM, Blumwald E, Mittler R. 2017. Reactive oxygen species, abiotic stress and stress combination. The Plant Journal 90, 856–867. 27801967 10.1111/tpj.13299

[CIT0033] Clark D , Durner J, Navarre DA, Klessig DF. 2000. Nitric oxide inhibition of tobacco catalase and ascorbate peroxidase. Molecular Plant-Microbe Interactions 13, 1380–1384. 11106031 10.1094/MPMI.2000.13.12.1380

[CIT0034] Coelho DR , Palma FR, Paviani V, Bonini MG. 2022. Nuclear-localized, iron-bound superoxide dismutase-2 antagonizes epithelial lineage programs to promote stemness of breast cancer cells via a histone demethylase activity. Proceedings of the National Academy of Sciences, USA 119, e2110348119. 10.1073/pnas.2110348119 PMC9303987 35858297

[CIT0035] Considine MJ , Foyer CH. 2021. Oxygen and reactive oxygen species (ROS) dependent regulation of plant growth and development. Plant Physiology 186, 79–92. 33793863 10.1093/plphys/kiaa077 PMC8154071

[CIT0036] Contreras-de la Rosa PA , Aragón-Rodríguez C, Ceja-López JA, García-Arteaga KF, De-la-Peña C. 2022. Lysine crotonylation: a challenging new player in the epigenetic regulation of plants. Journal of Proteomics 255, 104488. 35065287 10.1016/j.jprot.2022.104488

[CIT0037] Corpas FJ , Alché JD, Barroso JB. 2013. Current overview of S-nitrosoglutathione functions in plants. Frontiers in Plant Science 4, 126. 23658557 10.3389/fpls.2013.00126 PMC3647110

[CIT0038] Correa-Aragunde N , Foresi N, Lamattina L. 2015. Nitric oxide is a ubiquitous signal for maintaining redox balance in plant cells: regulation of ascorbate peroxidase as a case study. Journal of Experimental Botany 66, 2913–2921. 25750426 10.1093/jxb/erv073

[CIT0039] Couto N , Wood J, Barber J. 2016. The role of glutathione reductase and related enzymes on cellular redox homoeostasis network. Free Radical Biology & Medicine 95, 27–42. 26923386 10.1016/j.freeradbiomed.2016.02.028

[CIT0040] Das BK , Khan WA, Sreekumar SN, Ponraj K, Achary VMM, Reddy ES, Balasubramaniam D, Chandele A, Reddy MK, Arockiasamy A. 2023. Plant dehydroascorbate reductase moonlights as membrane integrated ion channel. Archives of Biochemistry and Biophysics 741, 109603. 37084805 10.1016/j.abb.2023.109603

[CIT0041] Das BK , Kumar A, Maindola P, Mahanty S, Jain SK, Reddy MK, Arockiasamy A. 2016. Non-native ligands define the active site of *Pennisetum glaucum* (L.) R. Br dehydroascorbate reductase. Biochemical and Biophysical Research Communications 473, 1152–1157. 27067046 10.1016/j.bbrc.2016.04.031

[CIT0042] Delaunay A , Pflieger D, Barrault MB, Vinh J, Toledano MB. 2002. A thiol peroxidase is an H_2_O_2_ receptor and redox-transducer in gene activation. Cell 111, 471–481. 12437921 10.1016/s0092-8674(02)01048-6

[CIT0043] Delorme-Hinoux V , Bangash SAK, Meyer AJ, Reichheld JP. 2016. Nuclear thiol redox systems in plants. Plant Science 243, 84–95. 26795153 10.1016/j.plantsci.2015.12.002

[CIT0044] de Pinto MC , Locato V, Sgobba A, Romero-Puertas Mdel C, Gadaleta C, Delledonne M, De Gara L. 2013. S-nitrosylation of ascorbate peroxidase is part of programmed cell death signaling in tobacco Bright Yellow-2 cells. Plant Physiology 163, 1766–1775. 24158396 10.1104/pp.113.222703 PMC3846137

[CIT0045] de Simone A , Hubbard R, de la Torre NV, Velappan Y, Wilson M, Considine MJ, Soppe WJJ, Foyer CH. 2017. Redox changes during the cell cycle in the embryonic root meristem of *Arabidopsis thaliana*. Antioxidants and Redox Signaling 27, 1505–1519. 28457165 10.1089/ars.2016.6959 PMC5678362

[CIT0046] Deslous P , Bournonville C, Decros G, et al. 2021. Overproduction of ascorbic acid impairs pollen fertility in tomato. Journal of Experimental Botany 72, 3091–3107. 33530105 10.1093/jxb/erab040

[CIT0047] Diao Y , Xu H, Li G, Yu A, Yu X, Hu W, Zheng X, Li S, Wang Y, Hu Z. 2014. Cloning a glutathione peroxidase gene from *Nelumbo nucifera* and enhanced salt tolerance by overexpressing in rice. Molecular Biology Reports 41, 4919–4927. 24715609 10.1007/s11033-014-3358-4

[CIT0048] Diaz-Vivancos P , de Simone A, Kiddle G, Foyer CH. 2015. Glutathione—linking cell proliferation to oxidative stress. Free Radical Biology & Medicine 89, 1154–1164. 26546102 10.1016/j.freeradbiomed.2015.09.023

[CIT0049] Ding H , Wang B, Han Y, Li S. 2020. The pivotal function of dehydroascorbate reductase in glutathione homeostasis in plants. Journal of Experimental Botany 71, 3405–3416. 32107543 10.1093/jxb/eraa107

[CIT0050] Dixon DP , Skipsey M, Grundy NM, Edwards R. 2005. Stress-induced protein S-glutathionylation in Arabidopsis. Plant Physiology 138, 2233–2244. 16055689 10.1104/pp.104.058917 PMC1183410

[CIT0051] Dorion S , Ouellet JC, Rivoal J. 2021. Glutathione metabolism in plants under stress: beyond reactive oxygen species detoxification. Metabolites 11, 641. 34564457 10.3390/metabo11090641 PMC8464934

[CIT0052] Dowdle J , Ishikawa T, Gatzek S, Rolinski S, Smirnoff N. 2007. Two genes in *Arabidopsis thaliana* encoding GDP-l-galactose phosphorylase are required for ascorbate biosynthesis and seedling viability. The Plant Journal 52, 673–689. 17877701 10.1111/j.1365-313X.2007.03266.x

[CIT0053] Dumanović J , Nepovimova E, Natić M, Kuča K, Jaćević V. 2021. The significance of reactive oxygen species and antioxidant defense system in plants: a concise overview. Frontiers in Plant Science 11, 552969. 33488637 10.3389/fpls.2020.552969 PMC7815643

[CIT0054] Eastmond PJ. 2007. Monodehydroascorbate reductase 4 is required for seed storage oil hydrolysis and postgerminative growth in Arabidopsis. The Plant Cell 19, 1376–1387. 17449810 10.1105/tpc.106.043992 PMC1913749

[CIT0055] Ehrary E , Rosas M, Carpinelli S, Davalos O, Cowling C, Fernandez F, Escobar M. 2020. Glutaredoxin *AtGRXS8* represses transcriptional and developmental responses to nitrate in *Arabidopsis thaliana* roots. Plants Direct 4, e00227. 10.1002/pld3.227 PMC7287413 32537558

[CIT0056] Eljebbawi H , del Carmen Rondón Guerrero Y, Dunand C, Estevez JM. 2021. Highlighting reactive oxygen species (ROS) as multitaskers in root development. iScience 24, 101978. 33490891 10.1016/j.isci.2020.101978 PMC7808913

[CIT0057] Eltayeb AE , Kawano N, Badawi GH, Kaminaka H, Sanekata T, Shibahara T, Inanaga S, Tanaka K. 2007. Overexpression of monodehydroascorbate reductase in transgenic tobacco confers enhanced tolerance to ozone, salt and polyethylene glycol stresses. Planta 225, 1255–1264. 17043889 10.1007/s00425-006-0417-7

[CIT0058] Eltelib H , Fujikawa Y, Esaka M. 2012. Overexpression of the acerola (*Malpighia glabra*) monodehydroascorbate reductase gene in transgenic tobacco plants results in increased ascorbate levels and enhanced tolerance to salt stress. South African Journal of Botany 78, 295–301.

[CIT0059] Faltin Z , Holland D, Velcheva M, Tsapovetsky M, Roeckel-Drevet P, Handa AK, Abu-Abied M, Friedman-Einat M, Eshdat Y, Perl A. 2010. Glutathione peroxidase regulation of reactive oxygen species level is crucial for in vitro plant differentiation. Plant and Cell Physiology 51, 2010. 10.1093/pcp/pcq082 20530511

[CIT0060] Fichman Y , Mittler R. 2020. Rapid systemic signaling during abiotic and biotic stresses: is the ROS wave master of all trades? The Plant Journal 102, 887–896. 31943489 10.1111/tpj.14685

[CIT0061] Fichman Y , Mittler R. 2021a. Integration of electric, calcium, reactive oxygen species and hydraulic signals during rapid systemic signaling in plants. The Plant Journal 107, 7–20. 34058040 10.1111/tpj.15360

[CIT0062] Fichman Y , Mittler R. 2021b. A systemic whole-plant change in redox levels accompanies the rapid systemic response to wounding. Plant Physiology 186, 4–8. 33793948 10.1093/plphys/kiab022 PMC8154084

[CIT0063] Fichman Y , Rowland L, Oliver MJ, Mittler R. 2023. ROS are evolutionary conserved cell-to-cell stress signals. Proceedings of the National Academy of Sciences, USA 120, e2305496120. 10.1073/pnas.2305496120 PMC10400990 37494396

[CIT0064] Fichman Y , Zandalinas SI, Peck S, Luan S, Mittler R. 2022. HPCA1 is required for systemic reactive oxygen species and calcium cell-to-cell signaling and plant acclimation to stress. The Plant Cell 34, 4453–4471. 35929088 10.1093/plcell/koac241 PMC9724777

[CIT0065] Foyer CH , Baker A, Wright M, Sparkes I, Mhamdi A, Schippers JHM, Van Breusegem F. 2020a. On the move: redox-dependent protein relocation. Journal of Experimental Botany 71, 620–631. 31421053 10.1093/jxb/erz330

[CIT0066] Foyer CH , Halliwell B. 1976. The presence of glutathione and glutathione reductase in chloroplasts: a proposed role in ascorbic acid metabolism. Planta 133, 21–25. 24425174 10.1007/BF00386001

[CIT0067] Foyer CH , Hanke G. 2022. ROS production and signalling in chloroplasts: cornerstones and evolving concepts. The Plant Journal 111, 642–661. 35665548 10.1111/tpj.15856 PMC9545066

[CIT0068] Foyer CH , Kyndt T, Hancock RD. 2020b. Vitamin C in plants: novel concepts, new perspectives and outstanding issues. Antioxidants & Redox Signaling 32, 463–485. 31701753 10.1089/ars.2019.7819

[CIT0069] Foyer CH , Mullineaux PM. 1998. The presence of dehydroascorbate and dehydroascorbate reductase in plant tissues. FEBS Letters 425, 528–529. 9563527 10.1016/s0014-5793(98)00281-6

[CIT0070] Foyer CH , Noctor G. 2011. Ascorbate and glutathione: the heart of the redox hub. Plant Physiology 155, 2–18. 21205630 10.1104/pp.110.167569 PMC3075780

[CIT0071] Foyer CH , Souriau N, Perret S, Lelandais M, Kunert KJ, Pruvost C, Jouanin L. 1995. Overexpression of glutathione reductase but not glutathione synthetase leads to increases in antioxidant capacity and resistance to photoinhibition in poplar trees. Plant Physiology 109, 1047–1057. 8552710 10.1104/pp.109.3.1047 PMC161408

[CIT0072] Fujiwara A , Shimura H, Masuta C, Sano S, Inukai T. 2013. Exogenous ascorbic acid derivatives and dehydroascorbic acid are effective antiviral agents against Turnip mosaic virus in *Brassica rapa*. Journal of General Plant Pathology 79, 198–204.

[CIT0073] Gallie DR. 2013. The role ofl-ascorbic acid recycling in responding to environmental stress and in promoting plant growth. Journal of Experimental Botany 64, 433–443. 10.1093/jxb/ers330 23162122

[CIT0074] Gao F , Chen J, Ma T, Li H, Wang N, Li Z, Zhang Z, Zhou Y. 2014. The glutathione peroxidase gene family in *Thellungiella salsuginea*: genome-wide identification, classification, and gene and protein expression analysis under stress conditions. International Journal of Molecular Sciences 15, 3319–3335. 24566152 10.3390/ijms15023319 PMC3958914

[CIT0075] García-Giménez JL , Romá-Mateo C, Pérez-Machado G, Peiró-Chova L, Pallardó FV. 2017. Role of glutathione in the regulation of epigenetic mechanisms in disease. Free Radical Biology & Medicine 112, 36–48. 28705657 10.1016/j.freeradbiomed.2017.07.008

[CIT0076] Gill SS , Anjum NA, Hasanuzzaman M, Gill R, Trivedi DK, Ahmad I, Pereira E, Tuteja N. 2013. Glutathione and glutathione reductase: a boon in disguise for plant abiotic stress defense operations. Plant Physiology and Biochemistry 70, 204–212. 23792825 10.1016/j.plaphy.2013.05.032

[CIT0077] González-Gordo S , Rodríguez-Ruiz M, López-Jaramillo J, Muñoz-Vargas MA, Palma JM, Corpas FJ. 2022. Nitric oxide (NO) differentially modulates the ascorbate peroxidase (APX) isozymes of sweet pepper (*Capsicum annuum* L.) fruits. Antioxidants 11, 765. 35453450 10.3390/antiox11040765 PMC9029456

[CIT0078] Gou JY , Li K, Wu K, et al. 2015. Wheat stripe rust resistance protein WKS1 reduces the ability of the thylakoid-associated ascorbate peroxidase to detoxify reactive oxygen species. The Plant Cell 27, 1755–1770. 25991734 10.1105/tpc.114.134296 PMC4498197

[CIT0079] Granlund I , Storm P, Schubert M, García-Cerdán JG, Funk C, Schröder WP. 2009. The TL29 protein is lumen located, associated with PSII and not an ascorbate peroxidase. Plant and Cell Physiology 50, 1898–1910. 19828564 10.1093/pcp/pcp134

[CIT0080] Gray B , Carmichael AJ. 1992. Kinetics of superoxide scavenging by dismutase enzymes and manganese mimics determined by electron spin resonance. The Biochemical Journal 281, 795–802. 1311175 10.1042/bj2810795 PMC1130760

[CIT0081] Grek CL , Zhang J, Manevich Y, Townsend DM, Tew KD. 2013. Causes and consequences of cysteine S-glutathionylation. Journal of Biological Chemistry 288, 26497–26504. 23861399 10.1074/jbc.R113.461368 PMC3772197

[CIT0082] Groden D , Beck E. 1979. H_2_O_2_ destruction by ascorbate-dependent systems from chloroplasts. Biochimica Biophysica Acta 546, 426435. 10.1016/0005-2728(79)90078-1 454577

[CIT0083] Gulyás Z , Székely A, Kulman K, Kocsy G. 2023. Light-dependent regulatory interactions between the redox system and miRNAs and their biochemical and physiological effects in plants. International Journal of Molecular Sciences 24, 8323. 37176028 10.3390/ijms24098323 PMC10179207

[CIT0084] Hasanuzzaman M , Nahar K, Anee TI, Fujita M. 2017. Glutathione in plants: biosynthesis and physiological role in environmental stress tolerance. Physiology and Molecular Biology of Plants 23, 249–268. 28461715 10.1007/s12298-017-0422-2 PMC5391355

[CIT0085] Hicks LM , Cahoon RE, Bonner RS, Rivard RS, Sheffield J, Jez JM. 2007. Thiol-based regulation of redox-active glutamate–cysteine ligase from *Arabidopsis thaliana*. The Plant Cell 19, 2653–2661. 17766407 10.1105/tpc.107.052597 PMC2002632

[CIT0086] Hong SH , Tripathi BN, Chung M-S, et al. 2018. Functional switching of ascorbate peroxidase 2 of rice (OsAPX2) between peroxidase and molecular chaperone. Scientific Reports 8, 9171. 29907832 10.1038/s41598-018-27459-1 PMC6003922

[CIT0087] Horemans N , Asard H, Caubergs RJ. 1994. The role of ascorbate free radical as an electron acceptor to cytochrome b-mediated trans-plasma membrane electron transport in higher plants. Plant Physiology 104, 1455–1458. 12232179 10.1104/pp.104.4.1455 PMC159312

[CIT0088] Horling F , Lamkemeyer P, König J, Finkemeier I, Kandlbinder A, Baier M, Dietz KJ. 2003. Divergent light-, ascorbate-, and oxidative stress-dependent regulation of expression of the peroxiredoxin gene family in Arabidopsis. Plant Physiology 131, 317–325. 12529539 10.1104/pp.010017 PMC166811

[CIT0089] Hossain MA , Nakano Y, Asada K. 1984. Monodehydroascorbate reductase in spinach chloroplasts and its participation in regeneration of ascorbate for scavenging hydrogen peroxide. Plant and Cell Physiology 25, 385–395.

[CIT0090] Hu Z , Li J, Ding S, Cheng F, Li X, Jiang Y, Yu J, Foyer CH, Shi K. 2021. The protein kinase CPK28 phosphorylates ascorbate peroxidase and enhances thermotolerance in tomato. Plant Physiology 186, 1302–1317. 33711164 10.1093/plphys/kiab120 PMC8195530

[CIT0091] Huang X , Chen S, Li W, et al. 2021. ROS regulated reversible protein phase separation synchronizes plant flowering. Nature Chemical Biology 17, 549–557. 33633378 10.1038/s41589-021-00739-0

[CIT0092] Igamberdiev AU , Bykova NV, Hill RD. 2006. Nitric oxide scavenging by barley hemoglobin is facilitated by a monodehydroascorbate reductase-mediated ascorbate reduction of methemoglobin. Planta 223, 1033–1040. 16341544 10.1007/s00425-005-0146-3

[CIT0093] Jardim-Messeder D , Zamocky M, Sachetto-Martins G, Margis-Pinheiro M. 2022. Chloroplastic ascorbate peroxidases targeted to stroma or thylakoid membrane: the chicken or egg dilemma. FEBS Letters 596, 2989–3004. 35776057 10.1002/1873-3468.14438

[CIT0094] Johnston EJ , Rylott EL, Beynon E, Lorenz A, Chechik V, Bruce NC. 2015. Monodehydroascorbate reductase mediates TNT toxicity in plants. Science 349, 1072–1075. 26339024 10.1126/science.aab3472

[CIT0095] Karpinska B , Zhang K, Rasool B, Pastok D, Morris J, Verrall SR, Hedley PE, Hancock RD, Foyer CH. 2017. The redox state of the apoplast influences the acclimation of photosynthesis and leaf metabolism to changing irradiance. Plant, Cell & Environment 41, 1083–1097. 10.1111/pce.12960 PMC5947596 28369975

[CIT0096] Kataya AR , Reumann S. 2010. Arabidopsis glutathione reductase 1 is dually targeted to peroxisomes and the cytosol. Plant Signaling & Behavior 5, 171–175. 20038819 10.4161/psb.5.2.10527 PMC2884127

[CIT0097] Kaur S , Prakash P, Bak DH, et al. 2021. Regulation of dual activity of ascorbate peroxidase 1 from *Arabidopsis thaliana* by conformational changes and posttranslational modifications. Frontiers in Plant Science 12, 678111. 34194454 10.3389/fpls.2021.678111 PMC8236860

[CIT0098] Kavitha K , George S, Venkataraman G, Parida A. 2010. A salt‐inducible chloroplastic monodehydroascorbate reductase from halophyte *Avicennia marina* confers salt stress tolerance on transgenic plants. Biochimie 92, 1321–1329. 20600571 10.1016/j.biochi.2010.06.009

[CIT0099] Kelly GJ , Latzko E. 1979. Soluble ascorbate peroxidase, detection in plants and use in vitamin C estimation. Naturwissenschaften 66, 617–619. 537642 10.1007/BF00405128

[CIT0100] Kerchev PI , Karpińska B, Morris JA, Hussain A, Verrall SR, Hedley PE, Fenton B, Foyer CH, Hancock RD. 2013. Vitamin C and the abscisic acid-insensitive 4 (ABI4) transcription factor are important determinants of aphid resistance in Arabidopsis. Antioxidants & Redox Signaling 18, 2091–2105. 23343093 10.1089/ars.2012.5097

[CIT0101] Keyster M , Klein A, Egbichi I, Jacobs A, Ludidi N. 2011. Nitric oxide increases the enzymatic activity of three ascorbate peroxidase isoforms in soybean root nodules. Plant Signaling & Behavior 6, 956–961. 21494099 10.4161/psb.6.7.14879 PMC3257769

[CIT0102] Kiddle G , Pastori GM, Bernard B, Pignocchi C, Antoniw J, Verrier PJ, Foyer CH. 2003. Effects of leaf ascorbate content on defense and photosynthesis gene expression in *Arabidopsis thaliana*. Antioxidants & Redox Signalling 5, 23–32. 10.1089/152308603321223513 12626114

[CIT0103] Kneeshaw S , Keyani R, Delorme-Hinoux V, Imrie L, Loake GJ, Le Bihan T, Reichheld JP, Spoel SH. 2017. Nucleoredoxin guards against oxidative stress by protecting antioxidant enzymes. Proceedings of the National Academy of Sciences, USA 114, 8414–8419. 10.1073/pnas.1703344114 PMC5547615 28724723

[CIT0104] Kotchoni SO , Larrimore KE, Mukherjee M, Kempinski CF, Barth C. 2009. Alterations in the endogenous ascorbic acid content affect flowering time in Arabidopsis. Plant Physiology 149, 803–815. 19028878 10.1104/pp.108.132324 PMC2633856

[CIT0105] Kumar RS , Shen C-H, Wu P-Y, Kumar S, Hua MS, Yeh K-Y. 2016. Nitric oxide participates in plant flowering repression by ascorbate. Science Reports 6, 35246. 10.1038/srep35246 PMC5059679 27731387

[CIT0106] Lazzarotto F , Turchetto-Zolet AC, Margis-Pinheiro M. 2015. Revisiting the non-animal peroxidase superfamily. Trends in Plant Science 20, 807–813. 26463217 10.1016/j.tplants.2015.08.005

[CIT0107] Lazzarotto F , Wahni K, Piovesana M, Maraschin F, Messens J, Margis-Pinheiro M. 2021. Arabidopsis APx-R is a plastidial ascorbate-independent peroxidase regulated by photomorphogenesis. Antioxidants 10, 65. 33430242 10.3390/antiox10010065 PMC7825652

[CIT0108] Le Gal K , Schmidt EE, Sayin VI. 2021. Cellular redox homeostasis. Antioxidants 10, 1377. 34573009 10.3390/antiox10091377 PMC8469889

[CIT0109] Li F , Wu QY, Sun YL, Wang LY, Yang XH, Meng QW. 2010. Overexpression of chloroplastic monodehydroascorbate reductase enhanced tolerance to temperature and methyl viologen‐mediated oxidative stresses. Physiologia Plantarum 139, 421–434. 20230481 10.1111/j.1399-3054.2010.01369.x

[CIT0110] Li S. 2023. Novel insight into functions of ascorbate peroxidase in higher plants: more than a simple antioxidant enzyme. Redox Biology 64, 102789. 37352686 10.1016/j.redox.2023.102789 PMC10333675

[CIT0111] Li Y , Dhankher OP, Carreira L, Balish RS, Meagher RB. 2005. Arsenic and mercury tolerance and cadmium sensitivity in Arabidopsis plants expressing bacterial γ-glutamylcysteine synthetase. Environmental Toxicology and Chemistry 24, 1386. 10.1897/04-340r.1 16117113

[CIT0112] Li Y , Huang F, Tao Y, et al. 2022. BcGR1.1, a cytoplasmic localized glutathione reductase, enhanced tolerance to copper stress in *Arabidopsis thaliana*. Antioxidants 11, 389. 35204271 10.3390/antiox11020389 PMC8869148

[CIT0113] Liebthal M , Maynard D, Dietz KJ. 2018. Peroxiredoxins and redox signaling in plants. Antioxidants & Redox Signaling 28, 609–624. 28594234 10.1089/ars.2017.7164 PMC5806080

[CIT0114] Lin CC , Jih PJ, Lin HH, Lin JS, Chang LL, Shen YH, Jeng ST. 2011. Nitric oxide activates superoxide dismutase and ascorbate peroxidase to repress the cell death induced by wounding. Plant Molecular Biology 77, 235–249. 21833542 10.1007/s11103-011-9805-x

[CIT0115] Lin P , Bai H, He, L Huang Q-R, Zeng Q-h, Pan Y-z, Jiang B-b, Zhang F, Zhang L, Liu Q-L. 2021. Proteome-wide and lysine crotonylation profiling reveals the importance of crotonylation in chrysanthemum (*Dendranthema grandiforum*) under low-temperature. BMC Genomics 22, 51. 33446097 10.1186/s12864-020-07365-5 PMC7809856

[CIT0116] Lisenbee CS , Lingard MJ, Trelease RN. 2005. Arabidopsis peroxisomes possess functionally redundant membrane and matrix isoforms of monodehydroascorbate reductase. The Plant Journal 43, 900–914. 16146528 10.1111/j.1365-313X.2005.02503.x

[CIT0117] Liso R , Innocenti AM, Bitonti MB, Arrigoni O. 1998. Ascorbic acid-induced progression of quiescent centre cells from G1 to S phase. New Phytologist 110, 469–471.

[CIT0118] Littler DR , Harrop SJ, Goodchild SC, et al. 2010. The enigma of the CLIC proteins: ion channels, redox proteins, enzymes, scaffolding proteins? FEBS Letters 584, 2093–2101. 20085760 10.1016/j.febslet.2010.01.027

[CIT0119] Liu F , Huang N, Wang L, et al. 2018. A novell-ascorbate peroxidase 6 gene, ScAPX6, plays an important role in the regulation of response to biotic and abiotic stresses in sugarcane. Frontiers in Plant Science 8, 2262. 10.3389/fpls.2017.02262 PMC5776131 29387074

[CIT0120] Liu J-X , Feng K, Duan A-Q, Li H, Yang Q-Q, Xu Z-S, Xiong A-S. 2019. Isolation, purification and characterization of an ascorbate peroxidase from celery and overexpression of the AgAPX1 gene enhanced ascorbate content and drought tolerance in Arabidopsis. BMC Plant Biology 19, 488–499. 31711410 10.1186/s12870-019-2095-1 PMC6849298

[CIT0121] Liu W , Barbosa Dos Santos I, Moye A, Park SW. 2020. CYP20-3 deglutathionylates 2-CysPRX A and suppresses peroxide detoxification during heat stress. Life Science Alliance 3, e202000775. 32732254 10.26508/lsa.202000775 PMC7409537

[CIT0122] Lu C , Jiang Y, Yue Y, et al. 2023. Glutathione and neodiosmin feedback sustain plant immunity. Journal of Experimental Botany 74, 976–990. 36346205 10.1093/jxb/erac442

[CIT0123] Mahmood AM , Dunwell JM. 2019. Evidence for novel epigenetic marks within plants. AIMS Genetics 6, 70–87. 31922011 10.3934/genet.2019.4.70 PMC6949463

[CIT0124] Mano E , Hideg K, Asada K. 2004. Ascorbate in thylakoid lumen functions as an alternative electron donor to photosystem II and to photosystem I. Archives of Biochemistry and Biophysics 429, 71–80. 15288811 10.1016/j.abb.2004.05.022

[CIT0125] Martins L , Knuesting J, Bariat L, et al. 2020. Redox modification of the iron–sulfur glutaredoxin GRXS17 activates holdase activity and protects plants from heat stress. Plant Physiology 184, 676–692. 32826321 10.1104/pp.20.00906 PMC7536686

[CIT0126] Marty L , Bausewein D, Müller C, et al. 2019. Arabidopsis glutathione reductase 2 is indispensable in plastids, while mitochondrial glutathione is safeguarded by additional reduction and transport systems. New Phytologist 224, 1569–1584. 31372999 10.1111/nph.16086

[CIT0127] Maruta T , Tanouchi A, Tamoi M, Yabuta Y, Yoshimura K, Ishikawa T, Shigeoka S. 2010. Arabidopsis chloroplastic ascorbate peroxidase isoenzymes play a dual role in photoprotection and gene regulation under photooxidative stress. Plant & Cell Physiology 51, 190–200. 20007290 10.1093/pcp/pcp177

[CIT0128] Mase K , Tsukagoshi H. 2021. Reactive oxygen species link gene regulatory networks during Arabidopsis root development. Frontiers in Plant Science 12, 660274. 33986765 10.3389/fpls.2021.660274 PMC8110921

[CIT0129] Mehlhorn H , Lelandais M, Korth HG, Foyer CH. 1996. Ascorbate is the natural substrate for plant peroxidases. FEBS Letters 378, 203–206. 8557101 10.1016/0014-5793(95)01448-9

[CIT0130] Meister A. 1994. Glutathione–ascorbic acid antioxidant system in animals. Journal of Biological Chemistry 269, 9397–9400. 8144521

[CIT0131] Meyer AJ , Dreyer A, Ugalde JM, Feitosa-Araujo E, Dietz KJ, Schwarzländer M. 2021. Shifting paradigms and novel players in Cys-based redox regulation and ROS signaling in plants—and where to go next. Biological Chemistry 402, 399–423. 33544501 10.1515/hsz-2020-0291

[CIT0132] Meyer Y , Belin C, Delorme-Hinoux V, Reichheld JP, Riondet C. 2012. Thioredoxin and glutaredoxin systems in plants: molecular mechanisms, crosstalks, and functional significance. Antioxidants & Redox Signaling 17, 1124–1160. 22531002 10.1089/ars.2011.4327

[CIT0133] Meyer Y , Siala W, Bashandy T, Riondet C, Vignols F, Reichheld JP. 2008. Glutaredoxins and thioredoxins in plants. Biochimica et Biophysica Acta 1783, 589–600. 18047840 10.1016/j.bbamcr.2007.10.017

[CIT0134] Mielecki J , Gawroński P, Karpiński S. 2020. Retrograde signaling: understanding the communication between organelles. International Journal of Molecular Sciences 21, 6173. 32859110 10.3390/ijms21176173 PMC7503960

[CIT0135] Miyaji T , Kuromori T, Takeuchi Y, et al. 2015. AtPHT4;4 is a chloroplast-localized ascorbate transporter in Arabidopsis. Nature Communications 6, 5928. 10.1038/ncomms6928 PMC4308718 25557369

[CIT0136] Miyake C , Asada K. 1994. Ferredoxin-dependent photoreduction of the monodehydroascorbate radical in spinach thylakoids. Plant and Cell Physiology 35, 539–549.

[CIT0137] Morell S , Follmann H, De Tullio M, Häberlein I. 1997. Dehydroascorbate and dehydroascorbate reductase are phantom indicators of oxidative stress in plants. FEBS Letters 414, 567–570. 9323037 10.1016/s0014-5793(97)01074-0

[CIT0138] Morell S , Follmann H, De Tullio M, Häberlein I. 1998. Dehydroascorbate reduction: the phantom remaining. FEBS Letters 425, 530–531. 9563528 10.1016/s0014-5793(98)00282-8

[CIT0139] Mukherjee M , Larrimore KE, Ahmed NJ, Bedick TS, Barghouthi NT, Traw MB, Barth C. 2010. Ascorbic acid deficiency in Arabidopsis induces constitutive priming that is dependent on hydrogen peroxide, salicylic acid, and the NPR1 gene. Molecular Plant-Microbe Interactions 23, 340–351. 20121455 10.1094/MPMI-23-3-0340

[CIT0140] Müller-Moulé P , Conklin PL, Niyogi KK. 2002. Ascorbate deficiency can limit violaxanthin de-epoxidase activity in vivo. Plant Physiology 128, 970–977. 11891252 10.1104/pp.010924 PMC152209

[CIT0141] Müller-Moulé P , Golan T, Niyogi KK. 2014. Ascorbate-deficient mutants of Arabidopsis grow in high light despite chronic photo-oxidative stress. Plant Physiology 133, 748–760. 10.1104/pp.103.032375 PMC389940 14963245

[CIT0142] Munne-Bosch S. 2005. The role of α-tocopherol in plant stress tolerance. Journal of Plant Physiology 162, 743–748. 16008098 10.1016/j.jplph.2005.04.022

[CIT0143] Munné-Bosch S , Queval G, Foyer CH. 2013. The impact of global change factors on redox signaling underpinning stress tolerance. Plant Physiology 161, 5–19. 23151347 10.1104/pp.112.205690 PMC3532280

[CIT0144] Muthuramalingam M , Matros A, Scheibe R, Mock HP, Dietz KJ. 2013. The hydrogen peroxide-sensitive proteome of the chloroplast in vitro and in vivo. Frontiers in Plant Science 4, 54. 23516120 10.3389/fpls.2013.00054 PMC3601327

[CIT0145] Naqvi S , Zhu C, Farre G, et al. 2009. Transgenic multivitamin corn through biofortification of endosperm with three vitamins representing three distinct metabolic pathways. Proceedings of the National Academy of Sciences, USA 106, 7762–7767. 10.1073/pnas.0901412106 PMC2683132 19416835

[CIT0146] Narendra S , Venkataramani S, Shen G, Wang J, Pasapula V, Lin Y, Kornyeyev D, Holaday AS, Zhang H. 2006. The Arabidopsis ascorbate peroxidase 3 is a peroxisomal membrane-bound antioxidant enzyme and is dispensable for Arabidopsis growth and development. Journal of Experimental Botany 57, 3033–3042. 16873450 10.1093/jxb/erl060

[CIT0147] Navrot N , Gelhaye E, Jacquot JP, Rouhier N. 2006. Identification of a new family of plant proteins loosely related to glutaredoxins with four CxxC motives. Photosynthesis Research 89, 71–79. 16915354 10.1007/s11120-006-9083-7

[CIT0148] Neubauer C , Yamamoto HY. 1992. Mehler-peroxidase reaction mediates zeaxanthin formation and zeaxanthin-related fluorescence quenching in intact chloroplasts. Plant Physiology 99, 1354–1361. 16669044 10.1104/pp.99.4.1354 PMC1080632

[CIT0149] Noctor G , Arisi A-CM, Jouanin L, Kunert KJ, Rennenberg H, Foyer CH. 1998. Glutathione: biosynthesis, metabolism and relationship to stress tolerance explored in transformed plants. Journal of Experimental Botany 49, 623–647.

[CIT0150] Noctor G , Foyer CH. 1998. Ascorbate and glutathione: keeping active oxygen under control. Annual Review of Plant Physiology and Plant Molecular Biology 49, 249–279. 10.1146/annurev.arplant.49.1.249 15012235

[CIT0151] Noctor G , Mhamdi A, Chaouch S, Han Y, Neukermans J, Marquez-Garcia B, Queval G, Foyer CH. 2012. Glutathione in plants: an integrated overview. Plant, Cell & Environment 35, 454–484. 10.1111/j.1365-3040.2011.02400.x 21777251

[CIT0152] Noctor G , Queval G, Gakière B. 2006. NAD(P) synthesis and pyridine nucleotide cycling in plants and their potential importance in stress conditions. Journal of Experimental Botany 57, 1603–1620. 16714307 10.1093/jxb/erj202

[CIT0153] Noshi M , Hatanaka R, Tanabe N, Terai Y, Maruta T, Shigeoka S. 2016. Redox regulation of ascorbate and glutathione by a chloroplastic dehydroascorbate reductase is required for high-light stress tolerance in Arabidopsis. Bioscience, Biotechnology, & Biochemistry 80, 870–877. 26927949 10.1080/09168451.2015.1135042

[CIT0154] Noshi M , Yamada H, Hatanaka R, Tanabe N, Tamoi M, Shigeoka S. 2017. Arabidopsis dehydroascorbate reductase 1 and 2 modulate redox states of ascorbate–glutathione cycle in the cytosol in response to photooxidative stress. Bioscience, Biotechnology, & Biochemistry 81, 523–533. 27852156 10.1080/09168451.2016.1256759

[CIT0155] Ojeda V , Pérez-Ruiz JM, Cejudo FJ. 2018. 2-Cys peroxiredoxins participate in the oxidation of chloroplast enzymes in the dark. Molecular Plant 11, 1377–1388. 30292682 10.1016/j.molp.2018.09.005

[CIT0156] Page M , Sultana N, Paszkiewicz K, Florance H, Smirnoff N. 2012. The influence of ascorbate on anthocyanin accumulation during high light acclimation in *Arabidopsis thaliana*: further evidence for redox control of anthocyanin synthesis. Plant, Cell & Environment 35, 388–404. 10.1111/j.1365-3040.2011.02369.x 21631536

[CIT0157] Panchuk II , Zentgraf U, Volkov RA. 2005. Expression of the Apx gene family during leaf senescence of *Arabidopsis thaliana*. Planta 222, 926–932. 16034597 10.1007/s00425-005-0028-8

[CIT0158] Paradiso A , de Pinto MC, Locato V, De Gara L. 2012. Galactone-γ-lactone-dependent ascorbate biosynthesis alters wheat kernel maturation. Plant Biology 14, 652–658. 22300256 10.1111/j.1438-8677.2011.00543.x

[CIT0159] Park JW , Piszczek G, Rhee SG, Chock PB. 2011. Glutathionylation of peroxiredoxin I induces decamer to dimers dissociation with concomitant loss of chaperon activity. Biochemistry 50, 3204–3210. 21401077 10.1021/bi101373h PMC3176717

[CIT0160] Park SW , Li W, Viehhauser A, et al. 2013. Cyclophilin 20-3 relays a 12-oxo-phytodienoic acid signal during stress responsive regulation of cellular redox homeostasis. Proceedings of the National Academy of Sciences, USA 110, 9559–9564. 10.1073/pnas.1218872110 PMC3677464 23671085

[CIT0161] Pascal S , Scalla R. 1999. Purification and characterization of a safener-induced glutathione S-transferase from wheat (*Triticum aestivum*). Physiologia Plantarum 106, 17–27.

[CIT0162] Pasternak T , Palme K, Paponov IA. 2020. Glutathione enhances auxin sensitivity in Arabidopsis roots. Biomolecules 10, 1550. 33202956 10.3390/biom10111550 PMC7697393

[CIT0163] Pastori GM , Kiddle G, Antoniw J, Bernard S, Veljovic-Jovanovic S, Verrier PJ, Noctor G, Foyer CH. 2003. Leaf vitamin C contents modulate plant defense transcripts and regulate genes that control development through hormone signaling. The Plant Cell 15, 939–951. 12671089 10.1105/tpc.010538 PMC152340

[CIT0164] Patterson K , Walters LA, Cooper AM, Olvera JG, Rosas MA, Rasmusson AG, Escobar MA. 2016. Nitrate-regulated glutaredoxins control Arabidopsis primary root growth. Plant Physiology 170, 989–999. 26662603 10.1104/pp.15.01776 PMC4734588

[CIT0165] Pavet V , Olmos E, Kiddle G, Mowla S, Kumar S, Antoniw J, Alvarez ME, Foyer CH. 2005. Ascorbic acid deficiency activates cell death and disease resistance responses in Arabidopsis. Plant Physiology 139, 1291–1303. 16244149 10.1104/pp.105.067686 PMC1283766

[CIT0166] Pellny TK , Locato V, Diaz Vivancos P, Markovic J, De Gara L, Pallardó FV, Foyer CH. 2009. Pyridine nucleotide cycling and control of intracellular redox state in relation to poly (ADP-ribose) polymerase activity and nuclear localisation of glutathione during exponential growth of Arabidopsis cells in culture. Molecular Plant 2, 442–456. 19825628 10.1093/mp/ssp008

[CIT0167] Peskin AV , Pace PE, Behring JB, Paton LN, Soethoudt M, Bachschmid MM, Winterbourn CC. 2016. Glutathionylation of the active site cysteines of peroxiredoxin 2 and recycling by glutaredoxin. Journal of Biological Chemistry 291, 3053–3062. 26601956 10.1074/jbc.M115.692798 PMC4742766

[CIT0168] Pignocchi C , Kiddle G, Hernández I, Foster SJ, Asensi A, Taybi T, Barnes J, Foyer CH. 2006. Ascorbate oxidase-dependent changes in the redox state of the apoplast modulate gene transcript accumulation leading to modified hormone signaling and orchestration of defense processes in tobacco. Plant Physiology 141, 423–435. 16603663 10.1104/pp.106.078469 PMC1475448

[CIT0169] Plumb W , Townsend AJ, Rasool B, Alomrani S, Razak N, Karpinska B, Ruban AV, Foyer CH. 2018. Ascorbate-mediated regulation of growth, photoprotection, and photoinhibition in *Arabidopsis thaliana*. Journal of Experimental Botany 69, 2823–2835. 29726917 10.1093/jxb/ery170 PMC5961140

[CIT0170] Pnueli L , Liang H, Rozenberg M, Mittler R. 2003. Growth suppression, altered stomatal responses, and augmented induction of heat shock proteins in cytosolic ascorbate peroxidase (Apx1)-deficient Arabidopsis plants. The Plant Journal 34, 187–203. 12694594 10.1046/j.1365-313x.2003.01715.x

[CIT0171] Rahantaniaina M , Tuzet A, Mhamdi A, Noctor G. 2013. Missing links in understanding redox signaling via thiol/disulfide modulation: how is glutathione oxidized in plants? Frontiers in Plant Science 4, 477. 24324478 10.3389/fpls.2013.00477 PMC3838956

[CIT0172] Rahantaniaina MS , Li S, Chatel-Innocenti G, Tuzet A, Issakidis-Bourguet E, Mhamdi A, Noctor G. 2017a. Cytosolic and chloroplastic DHARs cooperate in oxidative stress-driven activation of the salicylic acid pathway. Plant Physiology 174, 956–971. 28381499 10.1104/pp.17.00317 PMC5462045

[CIT0173] Rahantaniaina MS , Li S, Chatel-Innocenti G, Tuzet A, Mhamdi A, Vanacker H, Noctor G. 2017b. Glutathione oxidation in response to intracellular H_2_O_2_: key but overlapping roles for dehydroascorbate reductases. Plant Signaling & Behavior 12, e1356531. 28782990 10.1080/15592324.2017.1356531 PMC5616140

[CIT0174] Rai GK , Kumar P, Choudhary SM, et al. 2023. Antioxidant potential of glutathione and crosstalk with phytohormones in enhancing abiotic stress tolerance in crop plants. Plants 12, 1133. 36903992 10.3390/plants12051133 PMC10005112

[CIT0175] Ramakrishnan M , Papolu PK, Satish L, Vinod KK, Wei Q, Sharma A. Emamverdian A, Zou LH, Zhou M. 2022. Redox status of the plant cell determines epigenetic modifications under abiotic stress conditions and during developmental processes. Journal of Advanced Research 42, 99–116. 35690579 10.1016/j.jare.2022.04.007 PMC9788946

[CIT0176] Raven EL. 2003. Understanding functional diversity and substrate specificity in haem peroxidases: what can we learn from ascorbate peroxidase? Natural Product Reports 20, 367–381. 12964833 10.1039/b210426c

[CIT0177] Rey P , Taupin-Broggini M, Couturier J, Vignols F, Rouhier N. 2019. Is there a role for glutaredoxins and BOLAs in the perception of the cellular iron status in plants? Frontiers in Plant Science 10, 712. 31231405 10.3389/fpls.2019.00712 PMC6558291

[CIT0178] Rouhier N , Vlamis-Gardikas A, Lillig CH, Berndt C, Schwenn JD, Holmgren A, Jacquot JP. 2003. Characterization of the redox properties of poplar glutaredoxin. Antioxidants & Redox Signaling 5, 15–22. 12626113 10.1089/152308603321223504

[CIT0179] Saga G , Giorgetti A, Fufezan C, Giacometti GM, Bassi R, Morosinotto T. 2010. Mutation analysis of violaxanthin de-epoxidase identifies substrate-binding sites and residues involved in catalysis. Journal of Biological Chemistry 285, 23763–23770. 20507981 10.1074/jbc.M110.115097 PMC2911307

[CIT0180] Sakamoto A , Ueda M, Morikawa H. 2002. *Arabidopsis* glutathione-dependent formaldehyde dehydrogenase is an *S*-nitrosoglutathione reductase. FEBS Letters 515, 20–24. 11943187 10.1016/s0014-5793(02)02414-6

[CIT0181] Sakihama Y , Mano J, Sano S, Asada K, Yamasaki H. 2000. Reduction of phenoxyl radicals mediated by monodehydroascorbate reductase. Biochemical and Biophysical Research Communications 279, 949–954. 11162455 10.1006/bbrc.2000.4053

[CIT0182] Sankaranarayanan S , Ju Y, Kessler SA. 2020. Reactive oxygen species as mediators of gametophyte development and double fertilization in flowering plants. Frontiers in Plant Science 11, 1199. 32849744 10.3389/fpls.2020.01199 PMC7419745

[CIT0183] Seiml-Buchinger V , Reifschneider E, Bittner A, Baier M. 2022. Ascorbate peroxidase post-cold regulation of chloroplast NADPH dehydrogenase activity controls cold memory. Plant Physiology 190, 1997–2016. 35946757 10.1093/plphys/kiac355 PMC9614503

[CIT0184] Sevilla F , Martí MC, De Brasi-Velasco S, Jiménez A. 2023. Redox regulation, thioredoxins, and glutaredoxins in retrograde signalling and gene transcription. Journal of Experimental Botany 74, 5955–5969. 37453076 10.1093/jxb/erad270 PMC10575703

[CIT0185] Sha S , Minakuchi K, Higaki N, et al. 1997. Purification and characterization of glutaredoxin (thioltransferase) from rice (*Oryza sativa* L.). Journal of Biochemistry 121, 842–848. 9192723 10.1093/oxfordjournals.jbchem.a021663

[CIT0186] Shaikhali J , Baier M. 2016. Ascorbate regulation of 2-Cys peroxiredoxin-A promoter activity is light-dependent. Journal of Plant Physiology 167, 461–467. 10.1016/j.jplph.2009.10.021 20022402

[CIT0187] Shaikhali J , Heiber I, Seidel T, Ströher E, Hiltscher H, Birkmann S, Dietz KJ, Baier M. 2008. The redox-sensitive transcription factor Rap2.4a controls nuclear expression of 2-Cys peroxiredoxin A and other chloroplast antioxidant enzymes. BMC Plant Biology 8, 48. 18439303 10.1186/1471-2229-8-48 PMC2386467

[CIT0188] Shalata A , Mittova V, Volokita M, Guy M, Tal M. 2001. Response of the cultivated tomato and its wild salt-tolerant relative *Lycopersicon pennellii* to salt-dependent oxidative stress: the root antioxidative system. Physiologia Plantarum 112, 487–494. 11473708 10.1034/j.1399-3054.2001.1120405.x

[CIT0189] Shen CH , Krishnamurthy R, Yeh K. 2009. Decreasedl-ascorbate content mediating bolting is mainly regulated by the galacturonate pathway in *Oncidium*. Plant and Cell Physiology 50, 935–946. 10.1093/pcp/pcp045 19307192

[CIT0190] Shikanai T , Takeda T, Yamauchi H, Sano S, Tomizawa K, Yokota A, Shigeoka S. 1998. Inhibition of ascorbate peroxidase under oxidative stress in tobacco having bacterial catalase in chloroplasts. FEBS Letters 428, 47–51. 9645472 10.1016/s0014-5793(98)00483-9

[CIT0191] Smirnoff N. 2018. Ascorbic acid metabolism and functions: a comparison of plants and mammals. Free Radical Biology & Medicine 122, 116–129. 29567393 10.1016/j.freeradbiomed.2018.03.033 PMC6191929

[CIT0192] Som S , Raha C, Chatterjee IB. 1983. Ascorbic acid: a scavenger of superoxide radical. Acta Vitaminologica et Enzymologica 5, 243–250. 6324567

[CIT0193] Strand DD , Livingston AK, Satoh-Cruz M, Froehlich JE, Maurino VG, Kramer DM. 2015. Activation of cyclic electron flow by hydrogen peroxide in vivo. Proceedings of the National Academy of Sciences, USA 112, 5539–5544. 10.1073/pnas.1418223112 PMC4418880 25870290

[CIT0194] Ströher E , Millar AH. 2012. The biological roles of glutaredoxins. The Biochemical Journal 446, 333–348. 22928493 10.1042/BJ20112131

[CIT0195] Szechyńska-Hebda M , Lewandowska M, Witoń D, Fichman Y, Mittler R, Karpiński SM. 2022. Aboveground plant-to-plant electrical signaling mediates network acquired acclimation. The Plant Cell 34, 3047–3065. 35595231 10.1093/plcell/koac150 PMC9338792

[CIT0196] Tanaka M , Takahashi R, Hamada A, Terai Y, Ogawa T, Sawa Y, Ishikawa T, Maruta T. 2021. Distribution and functions of monodehydroascorbate reductases in plants: comprehensive reverse genetic analysis of *Arabidopsis thaliana* enzymes. Antioxidants 10, 1726. 34829597 10.3390/antiox10111726 PMC8615211

[CIT0197] Terai Y , Ueno H, Ogawa T, Sawa Y, Miyagi A, Kawai-Yamada M, Ishikawa T, Takanori Maruta T. 2020. Dehydroascorbate reductases and glutathione set a threshold for high-light-induced ascorbate accumulation. Plant Physiology 183, 112–122. 32205453 10.1104/pp.19.01556 PMC7210653

[CIT0198] Tóth SZ , Puthur JT, Nagy V, Garab G. 2009. Experimental evidence for ascorbate-dependent electron transport in leaves with inactive oxygen evolving complexes. Plant Physiology 149, 1568–1578. 19144767 10.1104/pp.108.132621 PMC2649403

[CIT0199] Tsukagoshi H , Busch W, Benfey PN. 2010. Transcriptional regulation of ROS controls transition from proliferation to differentiation in the root. Cell 143, 606–616. 21074051 10.1016/j.cell.2010.10.020

[CIT0200] Vanacker, HB, Carver TLW, Foyer CH. 1998. Pathogen-induced changes in the antioxidant status of the apoplast in barley leaves. Plant Physiology 117, 1103–1114. 9662553 10.1104/pp.117.3.1103 PMC34926

[CIT0201] Vanacker V , Guichard M, Bohrer A-S, Issakidis-Bourguet E. 2018. Redox regulation of monodehydroascorbate reductase by thioredoxin y in plastids revealed in the context of water stress. Antioxidants 7, 183. 30563207 10.3390/antiox7120183 PMC6316508

[CIT0202] Van Breusegem F , Foyer CH, Mann GE. 2018. Reactive oxygen species are crucial ‘pro-life’ survival signals in plants. Free Radical Biology & Medicine 122, 1–3. 29730380 10.1016/j.freeradbiomed.2018.04.582

[CIT0203] van Buer J , Cvetkovic J, Baier M. 2016. Cold regulation of plastid ascorbate peroxidases serves as a priming hub controlling ROS signaling in *Arabidopsis thaliana*. BMC Plant Biology 16, 163. 27439459 10.1186/s12870-016-0856-7 PMC4955218

[CIT0204] Vaseghi M-J , Chibani K, Telman W, Liebthal MF, Gerken M, Schnitzer H, Mueller SM, Dietz K-J. 2019. The chloroplast 2-cysteine peroxiredoxin functions as thioredoxin oxidase in redox regulation of chloroplast metabolism. eLife 7, e38194. 10.7554/eLife.38194 PMC6221545 30311601

[CIT0205] Veal EA , Toone WM, Jones N, Morgan BA. 2002. Distinct roles for glutathione S-transferases in the oxidative stress response in *Schizosaccharomyces pombe*. Journal of Biological Chemistry 277, 35523–35531. 12063243 10.1074/jbc.M111548200

[CIT0206] Venkatesh J , Park SW. 2014. Role ofl-ascorbate in alleviating abiotic stresses in crop plants. Botanical Studies 55, 38. 10.1186/1999-3110-55-38 PMC5432849 28510969

[CIT0207] Wang X , Hargrove MS. 2013. Nitric oxide in plants: the roles of ascorbate and hemoglobin. PLoS One 8, e82611. 24376554 10.1371/journal.pone.0082611 PMC3869716

[CIT0208] Wang G , Hu C, Zhou J, et al. 2019. Systemic root–shoot signaling drives jasmonate-based root defense against nematodes. Current Biology 29, 3430–3438.e4. 31588001 10.1016/j.cub.2019.08.049

[CIT0209] Wang Z , Sun J, Zu X, et al. 2022. Pseudouridylation of chloroplast ribosomal RNA contributes to low temperature acclimation in rice. New Phytologist 236, 1708–1720. 36093745 10.1111/nph.18479

[CIT0210] Waszczak C , Carmody M, Kangasjärvi J. 2018. Reactive oxygen species in plant signaling. Annual Review of Plant Biology 69, 209–236. 10.1146/annurev-arplant-042817-040322 29489394

[CIT0211] Watanabe M , Chiba Y, Hirai MY. 2021. Metabolism and regulatory functions of *O*-acetylserine, *S*-adenosylmethionine, homocysteine, and serine in plant development and environmental responses. Frontiers in Plant Science 12, 643403. 34025692 10.3389/fpls.2021.643403 PMC8137854

[CIT0212] Wei S , Zhang W, Fu R, Zhang Y. 2021. Genome-wide characterization of 2-oxoglutarate and Fe(II)-dependent dioxygenase family genes in tomato during growth cycle and their roles in metabolism. BMC Genomics 22, 126. 33602133 10.1186/s12864-021-07434-3 PMC7891033

[CIT0213] Willems P , Van Breusegem F, Huang J. 2021. Contemporary proteomic strategies for cysteine redoxome profiling. Plant Physiology 186, 110–124. 33793888 10.1093/plphys/kiaa074 PMC8154054

[CIT0214] Winterbourn CC. 2016. Revisiting the reactions of superoxide with glutathione and other thiols. Archives of Biochemistry and Biophysics 595, 68–71. 27095219 10.1016/j.abb.2015.11.028

[CIT0215] Xue L , Li S, Sheng H, Feng H, Xu S, An L. 2007. Nitric oxide alleviates oxidative damage induced by enhanced ultraviolet-B radiation in cyanobacterium. Current Microbiology 55, 294–301. 17700985 10.1007/s00284-006-0621-5

[CIT0216] Yang H , Mu J, Chen L, Feng J, Hu J, Li L, Zhou JM, Zuo J. 2015. S-nitrosylation positively regulates ascorbate peroxidase activity during plant stress responses. Plant Physiology 167, 1604–1615. 25667317 10.1104/pp.114.255216 PMC4378166

[CIT0217] Ye N , Zhang J. 2012. Antagonism between abscisic acid and gibberellins is partially mediated by ascorbic acid during seed germination in rice. Plant Signaling & Behavior 7, 563–565. 22516812 10.4161/psb.19919 PMC3419020

[CIT0218] Yeh HL , Lin TH, Chen CC, Cheng TX, Chang HY, Lee TM. 2019. Monodehydroascorbate reductase plays a role in the tolerance of *Chlamydomonas reinhardtii* to photooxidative stress. Plant and Cell Physiology 60, 2167–2179. 31198969 10.1093/pcp/pcz110

[CIT0219] Yin L , Wang S, Eltayeb AE, Uddin MI, Yamamoto Y, Tsuji W, Takeuchi Y, Tanaka K. 2010. Overexpression of dehydroascorbate reductase, but not monodehydroascorbate reductase, confers tolerance to aluminium stress in transgenic tobacco. Planta 231, 609–621. 19960204 10.1007/s00425-009-1075-3

[CIT0220] Yokochi Y , Fukushi Y, Wakabayashi K, Yoshida K, Hisabori T. 2021. Oxidative regulation of chloroplast enzymes by thioredoxin and thioredoxin-like proteins in *Arabidopsis thaliana*. Proceedings of the National Academy of Sciences, USA 118, 51. 10.1073/pnas.2114952118 PMC8713810 34907017

[CIT0221] Yoshida S , Tamaoki M, Shikano T, et al. 2006. Cytosolic dehydroascorbate reductase is important for ozone tolerance in *Arabidopsis thaliana*. Plant and Cell Physiology 47, 304–308. 16361320 10.1093/pcp/pci246

[CIT0222] Yousuf PY , Hakeem KUR, Chandna R, Ahmad P. 2012. Role of glutathione reductase in plant abiotic stress. In: Ahmad P, Prasad MNV, eds. Abiotic stress responses in plants. New York: Springer, 149–158.

[CIT0223] Yu X , Pasternak T, Eiblmeier M, et al. 2013. Plastid-localized glutathione reductase2-regulated glutathione redox status is essential for Arabidopsis root apical meristem maintenance. The Plant Cell 25, 4451–4468. 24249834 10.1105/tpc.113.117028 PMC3875729

[CIT0224] Zaffagnini M , Michelet L, Massot V, Trost P, Lemaire SD. 2008. Biochemical characterization of glutaredoxins from *Chlamydomonas reinhardtii* reveals the unique properties of a chloroplastic CGFS-type glutaredoxin. Journal of Biological Chemistry 283, 8868–8876. 18216016 10.1074/jbc.M709567200

[CIT0225] Zandalinas SI , Fichman Y, Devireddy AR, Sengupta S, Azad RK, Mittler R. 2020. . Systemic signaling during abiotic stress combination in plants. Proceedings of the National Academy of Sciences, USA 117, 13810–13820. 10.1073/pnas.2005077117 PMC7306788 32471943

[CIT0226] Zandalinas SI , Sengupta S, Fritschi FB, Azad RK, Nechushtai R, Mittler R. 2021. The impact of multifactorial stress combination on plant growth and survival. New Phytologist 230, 1034–1048. 33496342 10.1111/nph.17232 PMC8048544

[CIT0227] Zechmann B. 2011. Subcellular distribution of ascorbate in plants. Plant Signaling & Behavior 6, 360–363. 21350341 10.4161/psb.6.3.14342 PMC3142415

[CIT0228] Zechmann B , Stumpe M, Mauch F. 2011. Immunocytochemical determination of the subcellular distribution of ascorbate in plants. Planta 233, 1–12. 20872269 10.1007/s00425-010-1275-x PMC3015205

[CIT0229] Zeng J , Dong Z, Wu H, Tian Z, Zhao Z. 2017. Redox regulation of plant cell fate. The EMBO Journal 36, 2844–2855. 28838936 10.15252/embj.201695955 PMC5623875

[CIT0230] Zentgraf U , Andrade-Galan AG, Bieker S. 2022. Specificity of H_2_O_2_ signaling in leaf senescence: is the ratio of H_2_O_2_ contents in different cellular compartments sensed in Arabidopsis plants? Cellular & Molecular Biology Letters 27, 4. 34991444 10.1186/s11658-021-00300-w PMC8903538

[CIT0231] Zhang L , Huang J, Su S, et al. 2021. FERONIA receptor kinase-regulated reactive oxygen species mediate self-incompatibility in *Brassica rapa*. Current Biology 31, 3004–3016. 34015250 10.1016/j.cub.2021.04.060

[CIT0232] Zhang L , Wu M, Teng Y, Jia S, Yu D, Wei T, Chen C, Song W. 2019. Overexpression of the glutathione peroxidase 5 (RcGPX5) gene from *Rhodiola crenulata* increases drought tolerance in *Salvia miltiorrhiza*. Frontiers in Plant Science 9, 1950. 30687353 10.3389/fpls.2018.01950 PMC6333746

[CIT0233] Zhao Z , Wang S, Liu M, Hu D, Dong Z. 2023. Control of DNA demethylation by superoxide anion in plant stem cells. Research Square10.21203/rs.3.rs-3313783/v1. [Preprint]. 39266722

[CIT0234] Zhithovich A. 2020. Nuclear and cytoplasmic functions of vitamin C. Chemical Research in Toxicology 33, 2515–2526. 33001635 10.1021/acs.chemrestox.0c00348 PMC7572711

[CIT0235] Zhou F , Zheng B, Wang F, Cao A, Xie S, Chen X, Schick JA, Jin X, Li H. 2021. Genome-wide analysis of MDHAR gene family in four cotton species provides insights into fiber development via regulating AsA redox homeostasis. Plants 10, 227. 33503886 10.3390/plants10020227 PMC7912408

[CIT0236] Zhou Y , Ge S, Jin L, et al. 2019. A novel CO_2_‐responsive systemic signaling pathway controlling plant mycorrhizal symbiosis. New Phytologist 224, 106–116. 31087385 10.1111/nph.15917

[CIT0237] Zur I , Kopeć P, Surówka E, et al. 2021. Impact of ascorbate–glutathione cycle components on the effectiveness of embryogenesis induction in isolated microspore cultures of barley and triticale. Antioxidants 10, 1254. 34439502 10.3390/antiox10081254 PMC8389252

